# Composite Improved Algorithm Based on Jellyfish, Particle Swarm and Genetics for UAV Path Planning in Complex Urban Terrain

**DOI:** 10.3390/s24237679

**Published:** 2024-11-30

**Authors:** Qi Wang, Wenjun Yi

**Affiliations:** National Key Laboratory of Transient Physics, Nanjing University of Science and Technology, Nanjing 210094, China; wq_wangqi@njust.edu.cn

**Keywords:** jellyfish search algorithm, particle swarm algorithm, genetic algorithm, UAV path planning, complex urban terrain modelling, CEC2005 benchmark functions

## Abstract

Path planning technology is of great consequence in the field of unmanned aerial vehicles (UAVs). In order to enhance the safety, path smoothness, and shortest path acquisition of UAVs undertaking tasks in complex urban multi-obstacle environments, this paper proposes an innovative composite improvement algorithm that integrates the advantages of the jellyfish search algorithm and the particle swarm algorithm. The algorithm effectively overcomes the shortcomings of a single algorithm, including parameter setting issues, slow convergence rates, and a tendency to become trapped in local optima. Additionally, it enhances the path smoothness, which improves the path optimisation. This enhances the capacity of UAVs to optimise their paths in environments characterised by multiple obstacles. To evaluate the practical effectiveness of the algorithm, a three-dimensional complex city model was constructed for the purposes of the study, and an adaptation function was designed for the purpose of evaluation. The experimental evaluation of 23 benchmark functions, the simulation test of the 3D city model, and 100 repetitive experiments demonstrate that the composite improved algorithm has a considerable advantage over the other comparative algorithms regarding performance. It exhibits fast convergence, high accuracy, and both global and local search capabilities, which enable the effective planning of a UAV flight path and the maintenance of good stability. In comparison to traditional algorithms, the composite improved algorithm demonstrably reduces the flight time and the number of obstacle avoidance manoeuvres required by the UAV. It provides robust technical support for the path planning of the UAV in complex urban environments and facilitates the advancement and implementation of related technologies.

## 1. Introduction

Unmanned aerial vehicles (UAVs), with their excellent manoeuvrability, high level of safety, long life, and reliable operational performance, have achieved a wide and deep range of applications in a number of industries. As well as demonstrating its extraordinary power in the military sector, drone technology is also playing a key and indispensable role in the civilian sector, penetrating and optimising a wide range of social and environmental areas, including disaster prevention and mitigation, traffic monitoring, plant protection, energy patrols, and wildlife monitoring, to name but a few. As a consequence of the ongoing reform of airspace management at low altitudes, it has become a significant trend for unmanned aerial vehicles (UAVs) to enter this domain and undertake a range of diverse missions. However, the advent of a multitude of low-altitude UAVs also gives rise to the potential for adverse effects on ground facilities and public safety. In light of these considerations, the question of how to effectively manage and regulate the flight activities of these drones to ensure safety and efficiency has become a pressing issue [1]. In the complex low-altitude environment, careful planning of a safe and practical flight path for UAVs has become a key initiative to ensure smooth mission execution and flight safety. This not only ensures the effective completion of tasks by UAVs but also minimises the potential risks to the ambient environment and the safety of the public. Consequently, the advancement of efficient path planning techniques to cope with various challenges in low-altitude flight has become a crucial objective in current research.

Path planning represents a fundamental aspect of unmanned aerial vehicle (UAV) autonomous flight technology. Extensive research has been conducted in this field, both domestically and internationally, resulting in the proposal of numerous path planning algorithms and the accumulation of traditional methods that have reached a degree of maturity for application. Common path planning techniques include heuristic algorithms, mathematical optimisation algorithms, and artificial potential field methods. Each of these methods possesses distinctive characteristics and provides crucial support for UAVs to achieve efficient and safe autonomous flight in complex environments [2].

Significant advances have been made by researchers in the field of unmanned aerial vehicle (UAV) path planning. A number of research teams have put forward a range of innovative algorithms and techniques for different application scenarios and technical challenges, with the aim of enhancing the mission execution efficiency and path planning capability of UAVs in complex environments. Junxiao Xue et al. [3] (2024) put forth a deep reinforcement learning model that is driven by knowledge and data, with the objective of addressing the issue of the low learning efficiency associated with deep reinforcement learning methods when undertaking UAV gaming tasks in complex environments. The UAV gaming path planning problem was transformed into an optimisation problem and solved using a genetic algorithm, with the searched paths stored as expert knowledge in a pool of playback experience for deep reinforcement learning. Subsequently, the network structure was constructed using the TD3 algorithm to collect online experience and update the network parameters throughout the training programme. The experimental results demonstrate that the method achieves an improvement in the convergence speed and learning stability; furthermore, a notable enhancement was observed in the UAV’s performance with regard to the planning of flight paths within the game environment. In a recent study, Weisan Yan et al. [4] (2024) proposed an autonomous exploration method for UAV flight paths. This method was designed with the aim of solving the time-consuming problem of autonomous exploration path planning for existing UAVs in underground environments. The results of the experimental study demonstrated that the method was able to safely and effectively complete the exploration in the downhole environment while reducing the time required for single path planning. This improvement was significant when compared to the traditional GBPlanner algorithm based on occupancy raster maps and the RRT* algorithm. In a recent study, Chengliang Fang et al. [5] (2024) proposed a novel multi-intelligence flexible execution evaluation (MASAC) algorithm to address the challenge of collaborative path planning for heterogeneous multi-UAVs in dynamic and uncertain environments. To this end, a reinforcement learning environment was developed, considering UAV dynamics, heterogeneity, and safe obstacle avoidance requirements. Performance metrics, including the task completion rate, formation retention rate, and flight time, were designed to assess the algorithm’s capabilities and limitations. Qiuyi Gu and Dapeng Li [6] (2024) put forth an enhanced RRT algorithm with the objective of addressing the shortcomings of the traditional RRT algorithm in UAV path planning, namely the high search randomness, path redundancy, and inadequate path smoothing. The findings of the simulation study indicate that the enhanced RRT algorithm markedly reduces the average planning time and increases the probability of path planning success when compared to the traditional RRT and A* algorithms in narrow channels and complex obstacle environments. This validates the feasibility and efficacy of the suggested algorithm. In a study published in 2024, Xiaofeng Yuan et al. [7] put forth a methodology for the planning of infiltration paths for unmanned aerial vehicles (UAVs) in irregularly shaped regions. This approach was devised with the objective of addressing the path planning challenges that arise when UAVs are tasked with operations such as covert penetration, infiltration, and target localisation within urban settings. The experimental outcomes demonstrate that the algorithm is capable of devising paths with reduced length while simultaneously avoiding the locations of guard posts with a high degree of efficacy. Hao Hu et al. [8] (2024) and Qicheng Meng et al. [9] (2024) both enhanced and optimised the JADE algorithm with the objective of facilitating its application to the UAV path planning problem in a range of scenarios. The JADE algorithm, a variant of the differential evolutionary algorithm, has demonstrated excellent performance in optimisation and robustness in the context of both underwater glider and UAV path planning problems. This is achieved through the application of a multivariate strategy and adaptive parameter control.

In order to address the multifaceted challenges inherent to complex environments, a number of pioneering algorithms and solutions have been put forth by scholars in the field. These studies not only offer efficient path planning methods for multi-UAV collaborative tasks but also delve into the potential and limitations of UAVs in practical applications. In a recent study, R Shivgan et al. [10] (2022) proposed two offline multi-UAV path planning algorithms, namely DETACH and STEER, with the specific aim of addressing the issue of collision risk due to route crossing in multi-UAV missions. By analysing the waypoint coverage effect and revenue model, the authors found that the STEER algorithm performed better in high-density waypoint scenarios, significantly improving the efficiency and revenue of the mission. This indicates that the algorithm is a feasible candidate for practical application in multi-UAV missions. G Gugan and A Haque [11] (2023) adopted a different approach, focusing on key issues in path planning for autonomous UAVs. Specifically, the research focused on the development of efficient algorithms for the rapid calculation of feasible and energy-efficient routes, with the objective of preventing collisions. By reviewing a substantial body of highly cited literature, they provided a comprehensive overview of the current trends and limitations in path planning research. Furthermore, they identified potential avenues for future research aimed at developing more practical path planning tools. L. Radácsi et al. [12] (2022) proposed an inventory control model for logistics warehouses. This model addresses the challenges of inventory management in environments where satellite positioning or IoT solutions cannot be used. It accomplishes this by implementing the efficient movement and imaging of UAVs in warehouses through path planning theory. The implementation of this model improves the efficiency and accuracy of logistics management.

The following scholars conducted reviews of several studies on UAV path planning, with a particular focus on the development of improved approaches to enhance the efficiency and safety of UAV path planning in complex environments and application-specific scenarios. RA Saeed et al. [13] (2022) investigated the challenge of UAV path planning in complex terrains or unknown environments, with a specific emphasis on the selection of optimal paths in the presence of obstacles and the avoidance of collisions. A variety of population optimisation algorithms were analysed, and the advantages of the improved algorithms in accelerating the path planning process were verified. This provides a better solution for UAV applications in complex environments. In a recent study, Angelopoulos et al. [14] (2022) presented a prototype system, designated as the Drone Brush, which is designed to address the challenge of immersive interaction in unmanned aerial vehicle (UAV) path planning, particularly for collaborative photogrammetry and inspection tasks. The system employs Microsoft HoloLens 2 to facilitate the creation of UAV navigation paths in three-dimensional space through the use of gestures. The study by Xiongwei Huang et al. [15] (2023) proposed a systematic approach to path planning based on Building Information Modelling (BIM) models to conduct inspections of buildings within the Architecture, Engineering, and Construction (AEC) industry. Through a comprehensive process that encompasses path planning, virtual simulation, and actual flight, the researchers were able to achieve an efficient inspection of building surfaces. In contrast, Yiming Miao et al. [16] (2022) put forth a UAV swarm-based MEC node scheme to address the shortcomings of traditional fixed-base stations in complex terrains. The study demonstrates that by integrating global and local path planning, the efficiency and energy utilisation of offloading services can be significantly enhanced, making it particularly well-suited for smart city and industrial IoT applications. EV Vazquez-Carmona et al. [17] (2022) proposed a dedicated path planning method for spraying UAVs in their study on the automation of public area disinfection during the COVID-19 outbreak. By optimising the spraying model and path planning, the method successfully avoids collisions in low-altitude flights and improves disinfection efficiency, thereby demonstrating its potential application for public health. In a recent study, Quan Shao et al. [18] (2022) proposed an innovative unmanned aerial vehicle (UAV) path planning strategy that incorporates a global heuristic algorithm for risk mitigation and customer service optimisation. The fundamental premise of this approach is that it not only contemplates the potential for UAVs to collide with environmental elements, including buildings, pedestrians, and vehicles, during flight but also effectively assesses the likelihood of such collisions by accurately calculating the associated risks. Concurrently, the methodology also examines the attributes of the logistics service object in depth and quantifies the service benefits, thereby ensuring that the path planning process optimises logistics efficiency while enhancing customer satisfaction. In comparison to the conventional path planning approach that merely seeks the shortest flight distance, this solution exhibits notable advantages. The method accurately identifies the risk factors and customer distribution in complex urban environments while also reducing the potential costs and safeguarding service quality and efficiency. Consequently, this strategy delineates an economical and secure flight trajectory for unmanned aerial vehicles (UAVs) in logistics operations, thereby optimising cost-effectiveness. GK Tevyashov et al. [19] (2022) addressed the issue of optimising flight path planning based on the theory of minimising the sub-area coverage time when utilising multiple UAVs for farmland surveying. The optimised path planning algorithm is more efficient than the traditional method, which involves UAVs moving from one end of the farmland to the other in a straight line with a gradual offset. The study provides a more efficient path planning solution for multi-UAV cooperative operations.

This paper addresses the significant challenges posed by the complex and evolving urban terrain to unmanned aerial vehicle (UAV) path planning. To this end, it presents an innovative integration of three optimisation algorithms—the jellyfish algorithm, particle swarm algorithm, and genetic algorithm—to construct a composite enhanced algorithmic strategy. The essence of this strategy lies in the ingenious incorporation of the dynamic search mechanism of the jellyfish algorithm into the particle swarm algorithmic framework. This integration significantly enhances the algorithm’s global vision, facilitating a more flexible and efficient exploration of potential optimal paths. Concurrently, this integration effectively balances global search and local fine-tuning, preventing the algorithm from prematurely falling into the trap of local optimality and ensuring comprehensive and in-depth exploration of the global optimal solution. Moreover, in order to enhance the diversity and innovation of the algorithm in generating paths in complex environments, this algorithm also integrates the essence of the genetic algorithm, specifically crossover and mutation operations. The implementation of these genetic mechanisms not only enhances the diversity of solution sets during the iterative process of the algorithm but also endows the algorithm with enhanced local search and fine-tuning capabilities, which can continuously generate novel and adaptive flight path candidates and filter out optimal solutions through adaptation evaluation. Consequently, this series of meticulously devised enhancements not only markedly enhances the precision and operational efficiency of the path planning algorithm but also guarantees that the UAV is able to safely and efficiently plan and execute flight missions in complex and evolving urban terrains. This exemplifies the considerable potential and advantages of this composite algorithm in addressing practical path planning challenges.

The paper is structured as follows: Section 2 presents the system modelling, which aims to transform the complex urban environment into a mathematical problem by constructing a 3D topographic map and analysing the cost of UAV path planning to derive the expression of the fitness function. Section 3 introduces the principles of the three basic algorithms and provides a detailed account of the operational procedures of the improved composite algorithm. Section 4 comprises two simulation experiments. The first tests the improved composite algorithm in conjunction with five comparative algorithms against the CEC2005 benchmark function, with the objective of verifying its global development and local search capability. The second experiment is designed to verify the actual path planning on 3D terrain maps, with the aim of demonstrating the rationality and effectiveness of the improved composite algorithm. Furthermore, to eliminate the possibility of erroneous results, 100 repetitions of the experiments were conducted to test the stability of the algorithm. Section 5 provides a concise overview of the study’s key findings and outlines the proposed research plan for subsequent investigations.

## 2. System Modelling

The urban environment presents a distinctive set of challenges relative to the field environment. These include the presence of high buildings above ground, underground pipelines, limited manoeuvre paths, complex electromagnetic conditions, and a concentration of civilians. Traditional platforms equipped by troops are particularly difficult to deploy under urban environmental conditions, and their effectiveness is significantly reduced in such an environment [20]. Small rotary-wing unmanned aerial vehicles (UAVs) are distinguished by their ability to take off and land vertically, their manoeuvrability, superior flight control, and platform versatility, which collectively afford them a distinct advantage when undertaking missions in urban environments. The ability to abstractly model buildings and airborne threats is a critical factor influencing the efficacy of path planning algorithms and the quality of resulting outputs. It can, therefore, be seen that the adoption of a reasonable environment modelling method can effectively simplify the path planning problem, thereby enhancing the algorithm’s performance and the reliability of the planning outcomes. In this work, we utilised a cost function to evaluate the multifactorial impact of path selection, thereby ensuring that path planning is both efficient and safe while maintaining a reasonable length. By quantifying the cost of different factors, it is possible to provide more accurate guidance for the path decision of UAVs in complex environments.

### 2.1. Environmental Model

**Figure 1:**
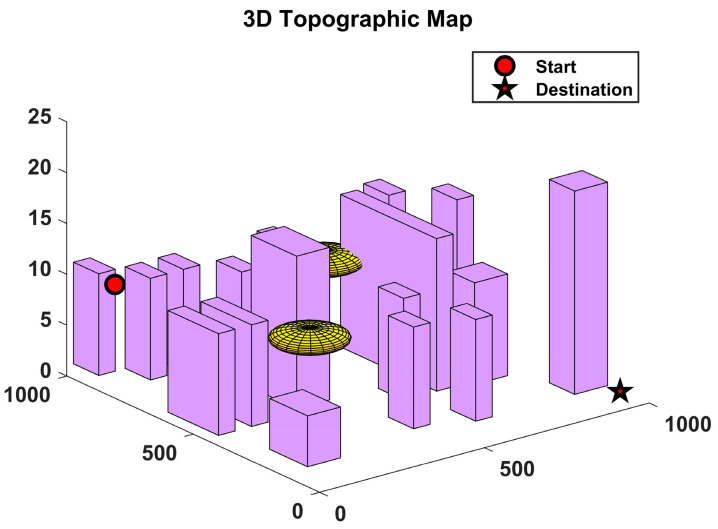
Three-dimensional topographic map.

In order to demonstrate the implementation of 3D spatial path planning algorithms, this work utilised simple geometric models, such as cubes and floating balls, to represent the threat’s influence range [21]. This approach effectively simplified the threat assessment in complex environments, as illustrated in Figure 1. In practical situations, it is often challenging to ascertain the precise coordinates of an obstacle’s geometric centre. To minimise the potential for inaccuracy, it is essential to expand the model to a sufficient scale to encompass the actual obstacle, thereby enhancing the security and precision of the path planning process.

**Figure 2:**
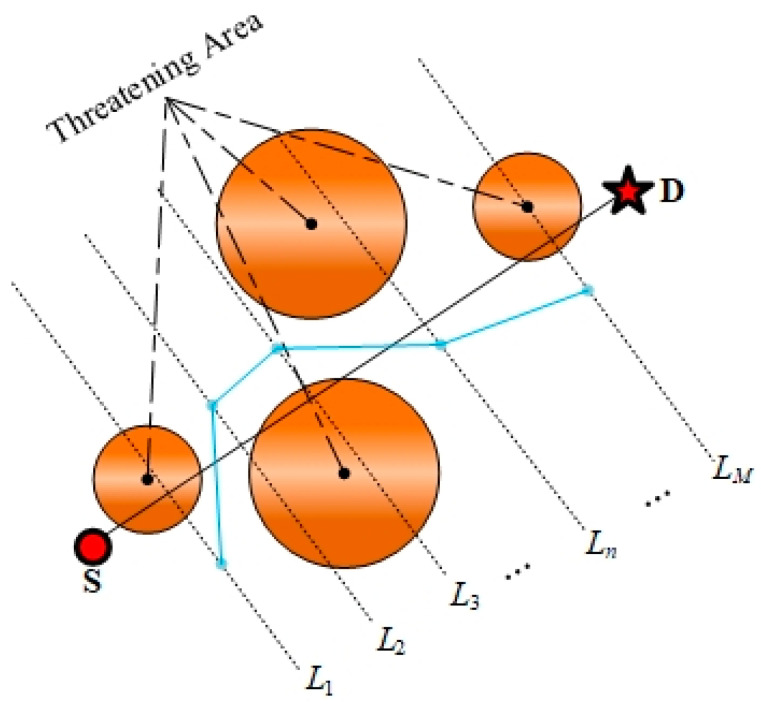
Schematic diagram of a two-dimensional path.

It is common practice to consider three-dimensional space as a stacked structure of two-dimensional space. Figure 2 illustrates a graphical representation of the two-dimensional approach to unmanned aerial vehicle (UAV) path planning. In this illustration, S and D signify the initial and final locations, respectively, in the path planning process, $L_{n}$ denotes the nth contour, and M designates the upper limit on the quantity of mission path contours that can be considered, 1≤n≤M.

To accelerate the search for an approximate optimal solution, the line segment SD can be partitioned into equal parts. At each equidistant point, a straight line perpendicular to SD is constructed, and a collection of discrete points Q is gathered on each perpendicular line $L_{n}$, thereby enhancing the resolution of the search space,

(1) $$Q=\left|S,L_{1}(x_{1},y_{1}),L_{2}(x_{2},y_{2}),\ldots ,L_{n}(x_{n},y_{n}),\ldots L_{M}(x_{M},y_{M}),D\right|.$$

**Figure 3:**
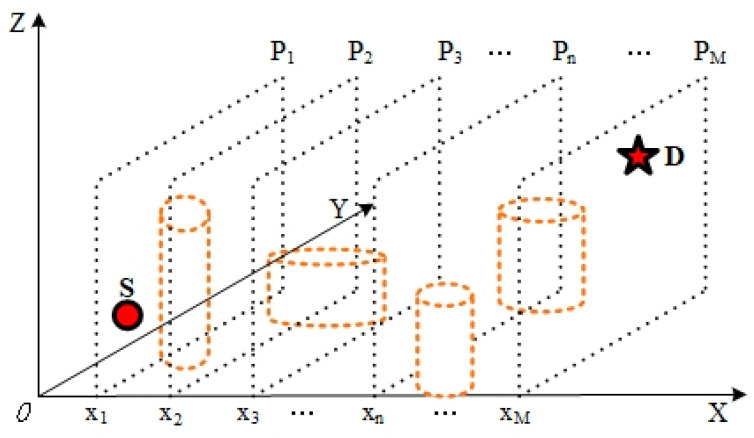
Schematic diagram of a two-dimensional path.

As with the two-dimensional treatment, planes perpendicular to the *X*-axis are created at equal points on the X-axis in three-dimensional space $\left(P_{1},P_{2},\ldots ,P_{M}\right)$. In order to obtain a set RD of discrete points in three-dimensional space, it is necessary to take a discrete point on each perpendicular plane, as illustrated in Figure 3.

(2) $$RD=\left|S,(x_{1},y_{1},z_{1}),\ldots ,(x_{n},y_{n},z_{n}),\ldots ,D\right|$$

Furthermore, the line segment SD is regarded as the X-axis, and a coordinate transformation (Equation (1)) is applied to each discrete point $\left(x_{n},y_{n}\right)$ [22]. In this equation, the angle θ is defined as the angle formed between the X-axis and the line segment connecting the start point S to the end point D, while $\left(x_{s},y_{s}\right)$ represent the specific coordinates of the various task take-off points within the same original coordinate system.

(3) $$\left[\begin{array}{l} x_{n}^{\prime }\\ y_{n}^{\prime } \end{array}\right]=\left[\begin{array}{c} \cos\theta \\ -\sin\theta \end{array}\right]\left[\begin{array}{l} x_{n}-x_{s}\\ y_{n}-y_{s} \end{array}\right]$$

In order to transform the coordinates $\left(x_{k},y_{k},z_{k}\right)$ of any given point within a three-dimensional space, the following formula is employed:

(4) $$\left[\begin{array}{l} x_{n}^{\prime }\\ y_{n}^{\prime }\\ z_{n}^{\prime } \end{array}\right]=\left[\begin{array}{ccc} \cos\theta & \sin\theta & 0\\ -\sin\theta & \cos\theta & 0\\ 0 & 0 & 1 \end{array}\right]\left[\begin{array}{l} x_{n}-x_{s}\\ y_{n}-y_{s}\\ z_{n}-z_{s} \end{array}\right].$$

Accordingly, the X-axis coordinates of all discrete points can be determined by means of a straightforward equation:

(5) $$x_{n}^{\prime }=\frac{\left|SD\right|}{M+1}.$$

Subsequently, the set of discrete points can be simplified as follows:

(6) $$C^{\prime }=\left\{0,L_{1}(y_{1}^{\prime }),L_{2}(y_{2}^{\prime }),\ldots ,L_{n}(y_{n}^{\prime }),\ldots ,L_{M}(y_{M}^{\prime })\right\}.$$

To alleviate the computational overhead, the present work introduces a novel technique of incorporating a rotating coordinate system along with evenly distributed coordinate axes for modelling threat sources. This innovation simplifies the path planning optimisation by reframing it as a streamlined optimisation of coordinate sequences. Consequently, identifying the optimal outcome of the objective function, i.e., the peak fitness value, becomes more straightforward. The application of this method boosts computational efficiency and endows the optimisation process with enhanced flexibility and adaptability.

### 2.2. Modelling of Obstacles and Threat Zones

Modelling obstacles is a crucial step in the path planning for unmanned aerial vehicles (UAVs). The manner in which obstacles are described has an impact on both the trajectory path representation and the search algorithm. In the planning space, the starting point is designated as S and the target point as D. As the unmanned aerial vehicle (UAV) progresses from its point of departure to its intended destination, it may encounter a multitude of common obstacles along the way. For the sake of the argument, let us assume that the threat zone is a cylindrical model [23]. The centre coordinate of the ith threat zone is $O_{i}(x_{i},y_{i})$, and the radius is $r_{i}$. These obstacles and threat zones can be represented by the matrix K as follows:

(7) $$K=\left[\begin{array}{ccc} x_{1} & y_{1} & r_{1}\\ x_{2} & y_{2} & r_{2}\\ \vdots & \vdots & \vdots \\ x_{i} & y_{i} & r_{i} \end{array}\right].$$

In this system of coordinates, the horizontal coordinate is represented by x, the vertical coordinate by y, and the threat radius by r. The threat height, represented by z, is the three-dimensional height of each obstacle. The value Z is represented by the vector $Z=[z_{1},z_{2},\ldots ,z_{i}]$.

### 2.3. Cost of Drone Paths

The terrain constraint cost function is employed to evaluate the constraints imposed by the terrain on flight paths, whereas the obstacle threat cost function is utilised to assess the impact of obstacles on the safety of flight. The flight distance cost function is designed to assess the influence of the flight distance on the efficiency, while the flight altitude cost function evaluates the impact of different flight altitudes on flight safety and efficiency. Additionally, the corner constraint cost function is employed to analyse the angular impact of turns. By integrating these cost functions, the effect of diverse factors on the flight path can be comprehensively evaluated, facilitating the development of more optimal path planning strategies.

Accordingly, a comprehensive cost function is constructed, integrating several key elements, including the terrain constraint cost, obstacle threat cost, flight distance cost, flight height adjustment cost, and corner constraint cost. The objective of this function is to evaluate the effectiveness and safety of flight paths in a comprehensive manner. The primary objective is to ascertain a flight trajectory that represents the optimal balance between cost-effectiveness and operational feasibility. This is achieved by meticulously calculating and aggregating all pertinent cost elements. In summary, the aim is to identify a path through the optimisation algorithm that minimises the overall flight cost while satisfying all the constraints, thus ensuring the economy and safety of the flight [24].

#### 2.3.1. Topographic Constraint Costs

In order to prevent unmanned aerial vehicles (UAVs) from colliding with the terrain during their missions, it is necessary to ensure that the planned routes are kept above the terrain altitude. When the flight altitude is lower or equal to the terrain altitude, the corresponding cost will be increased. This adjustment is designed to ensure the safety and effectiveness of the flight. The cost function is as follows:

(8) $$D_{i}=\left\{\begin{array}{l} 0,H_{threat}<H_{i}\\ (H_{threat}-H_{i})\times p_{1},else \end{array}\right.$$

(9) $$D=\sum_{i=1}^{m}D_{i}.$$

In this model, the cost of the terrain constraint generated by the UAV at a given path point $\left(x_{i},y_{i}\right)$ is represented by $D_{i}$. The terrain function is represented by $H_{threat}$, the UAV flight altitude is represented by $H_{i}$, the penalty value is represented by $p_{1}$, the total value of the terrain constraint cost generated by the path is represented by D, and the number of points on the path that the flight passes through is represented by m.

#### 2.3.2. Obstacle Threat Costs

In the realm of route planning, the optimisation of the path length is a key objective, as it enhances efficiency. However, ensuring the safe operation of unmanned aerial vehicles (UAVs) is equally important. To this end, we introduce the key element of obstacle threat cost, which enables the accurate assessment of the risk of obstacles encountered by the UAV during flight. This cost mechanism guides the UAV to automatically avoid all kinds of obstacles in the actual operating environment when planning its path. By calculating and incorporating the cost of obstacle threats, our path planning system generates economical and safe flight paths, effectively guaranteeing the safety and reliability of UAVs during mission execution.

**Figure 4:**
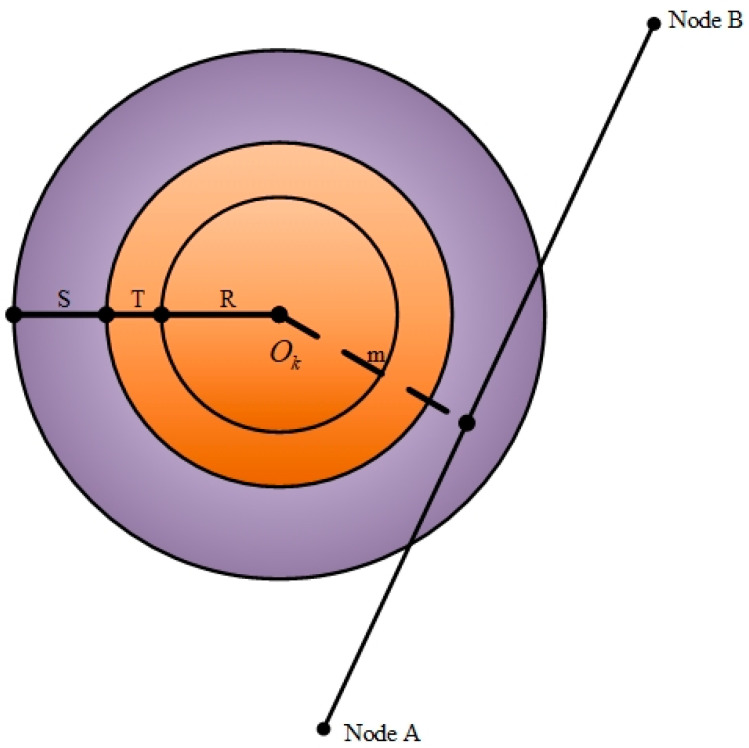
Obstacle schematic.

A total of M obstacles was set during the flight, with the square building acting as a cylinder to simulate the obstacles encountered. Concurrently, the size of the UAV was considered a circle with a diameter T. The coordinates of the projected centre point of each obstacle were denoted by $O_{m}$, with radius $r_{m}$, as illustrated in Figure 4.

For a given path segment from node 1 to node 2, the safe distance to fly is S, and the perpendicular distance between two neighbouring path nodes and $O_{m}$ is P. This indicates that the unmanned aerial vehicle (UAV) must remain within an area that is not obscured by shadows in order to ensure the safety of its operation.

At this point in the analysis, the obstacle threat cost for path i is represented by the quantity Q, where

(10) $$Q=\left\{\begin{array}{ll} \sum_{j=1}^{n-1}\sum_{M=1}^{M_{\max}}(S+T+r_{m})-P & \;T+R<P\leq S+T+r_{m}\\ 0 & P\;>S+T+r_{m}\\ \infty & P\leq T+r_{m} \end{array}\right..$$

#### 2.3.3. Flight Distance Cost

To optimise the energy usage of the unmanned aerial vehicle (UAV) during its flight, identifying the most efficient and shortest possible route is essential. Such an approach may enhance the efficiency of the flight and prolong its duration. The coordinates of point i on the UAV flight path in the 3D coordinate system xyz are $\left(x_{i},y_{i},z_{i}\right)$; thus, the distance between two successive points, specifically point i and point i−1, along the unmanned aerial vehicle’s flight trajectory is defined by the following formula:

(11) $$L(i)=\sqrt{{\left(x_{i}-x_{i-1}\right)}^{2}+{\left(y_{i}-y_{i-1}\right)}^{2}+{\left(z_{i}-z_{i-1}\right)}^{2}}.$$

The total distance traversed by the unmanned aerial vehicle (UAV) over the course of its entire flight path can be expressed as follows:

(12) $$L=L_{ss}+\sum_{i=2}^{m}L(i)+L_{dd}.$$

In this scenario, the variable m represents the total number of locations or points that the unmanned aerial vehicle (UAV) traverses during its journey. In particular, $L_{ss}$ denotes the distance between the UAV’s initial take-off point and its first path point, whereas $L_{dd}$ represents the distance between the UAV’s ultimate destination and the last path point it traverses.

#### 2.3.4. Flight Altitude Cost

It is of paramount importance to maintain a stable altitude during the flight of a UAV in order to guarantee flight stability and safety. In order to offset the adverse effects of sudden altitude shifts on the UAV’s operational efficacy and the successful completion of its mission, this work proposes the integration of flight altitude costs into the path planning process. This is achieved by quantifying the total altitude variation across the flight path, thereby facilitating the identification of optimal flight trajectories. By meticulously calculating the altitude differences between different segments of the UAV, the objective is to guide the UAV to select those flight trajectories that can maintain a relatively constant altitude. This results in a reduction in unnecessary lifting and lowering manoeuvres, which in turn enhances the smoothness and efficiency of flight. Such a design not only saves energy but also significantly reduces the risk of excessive altitude fluctuations and ensures that the UAV maintains an optimal flight state throughout the mission. The cost function is as follows:

(13) $$H(i)=\left|Z_{i}-Z_{i-1}\right|$$

(14) $$H=H_{ss}+\sum_{i=2}^{m}H(i)+H_{dd},$$

where the variable m denotes the total number of points passed by the unmanned aerial vehicle (UAV) during its flight. The term $H_{ss}$ denotes the altitude difference between the UAV’s initial point of departure and the first path point, while $H_{dd}$ signifies the altitude difference between the endpoint of the UAV’s mission objective and the final path point.

#### 2.3.5. Corner Constraint Cost

It is frequently necessary to make turns during the flight of a UAV due to environmental factors and other reasons. The maximum permissible turning angle is defined as the maximum deflection angle. A reduction in the deflection angle will result in a reduction in the turning amplitude of the UAV, thereby increasing the smoothness of the flight. Conversely, an increase in the deflection angle will result in a larger turning radius, thereby reducing the stability of the unmanned aerial vehicle (UAV). The corner cost function is designed as follows:

(15) $$\arctan\theta_{i}=\arctan\left(\frac{y_{i}-y_{i-1}}{x_{i}-x_{i-1}}\right)$$

(16) $$\Phi =\left|\arctan\theta_{i}-\arctan\theta_{i-1}\right|.$$

In this context, the variable $\theta_{i}$ represents the angular distance between two consecutive segments.

#### 2.3.6. Composite Cost Function

In conclusion, the integrated cost function is of paramount importance in the field of unmanned aerial vehicle (UAV) path planning. It provides a comprehensive and systematic evaluation of the integrated performance of the entire flight path, including all path points. This function considers not only the length efficiency of the flight path and the stability and safety of the flight altitude but also analyses the effects of terrain constraints, obstacle threats, and corner constraints on the flight performance in multiple dimensions in depth. By accurately calculating and integrating the costs or expenses of these key elements, the comprehensive cost function can provide a clear and quantitative index for measuring the advantages and disadvantages of different flight paths. Therefore, in the path planning process, the objective is to identify the flight path that minimises the integrated cost function, thereby achieving an optimal balance between flight efficiency, safety, stability, and other relevant performance metrics. The comprehensive cost function, designated as F, can be formulated as follows:

(17) F=D+Q+L+H+Φ.

In this context, the variables D, Q, L, H, and Φ represent the terrain constraint cost, obstacle threat cost, flight distance cost, flight height cost, and corner constraint cost, respectively.

This integrated cost function is employed as a fitness function to provide the algorithm with a transparent optimisation objective, thereby guiding the algorithm to continuously adjust the particles and gradually eliminate individuals with higher fitness, that is to say, paths with a lower integrated cost, within the population. Thus, this provides substantial support for achieving a superior path search.

## 3. Algorithm Design

There are a number of compelling reasons for combining particle swarm algorithms, genetic algorithms, and jellyfish search algorithms to address the path planning problem of unmanned aerial vehicles (UAVs). Firstly, it is evident that a single algorithm is subject to inherent limitations. The particle swarm algorithm is relatively simple, straightforward to implement, and exhibits a rapid convergence rate; however, it is susceptible to converging on local optimal solutions. A genetic algorithm is capable of conducting a comprehensive search of the global solution space; yet, its convergence speed is relatively slow. The jellyfish search algorithm has a distinctive search mechanism that can more effectively balance exploration and exploitation. However, the algorithm may exhibit unstable search behaviour in certain instances [25]. Secondly, the hybrid algorithm exhibits notable advantages. The combination of these three algorithms allows each to leverage its respective strengths, thereby greatly enhancing the overall search capability. The global search capability of the genetic algorithm can prevent the algorithm from falling into a local optimum. The fast convergence property of the particle swarm algorithm can accelerate the search process. Furthermore, the balanced exploration and exploitation capability of the jellyfish search algorithm can facilitate the exploration of complex path spaces. Consequently, the UAV is able to identify the optimal or near-optimal path in a more efficient manner within complex environments, thereby markedly enhancing the efficacy and success rate of mission execution. Furthermore, the hybrid algorithm can also enhance the stability and reliability of the algorithm.

### 3.1. Jellyfish Search Algorithm

The jellyfish search algorithm, introduced in 2021, represents a novel approach to swarm intelligence optimisation. Its conceptual foundation is the behaviour of jellyfish as they navigate the oceans in search of food, and it employs this natural phenomenon to construct a mathematical model that mimics their social dynamics. Its mathematical model simulates the social behaviour of these creatures. The simulation of jellyfish searching behaviour encompasses their current trajectory, their movements within the jellyfish swarm (active and passive), and a temporal control mechanism to switch between these movements. The proposed algorithm is based on three idealised rules: firstly, that jellyfish follow currents or jellyfish swarms; secondly, that the switching between the two modes of motion is controlled by a temporal control mechanism; and thirdly, that jellyfish are more biased towards places with high amounts of food in their search for food. Furthermore, the amount of food found is determined by the jellyfish position and its corresponding objective function. When compared with alternative heuristic methods, the jellyfish search algorithm demonstrates remarkable efficacy while requiring a minimal number of evaluations of the objective function, thereby illustrating its superiority in terms of efficiency and performance. It is distinguished by a robust capacity to identify optimal solutions and exhibits rapid convergence [26].

#### 3.1.1. Population Initialisation

The inception of the jellyfish swarm’s first generation is facilitated by the application of logistic chaotic mapping, where the logistic mapping itself is mathematically defined in the subsequent manner:

(18) $$Z_{i+1}=\eta Z_{i}(1-Z_{i}),0<Z_{0}<1.$$

The jellyfish swarm is constituted by a defined population size, designated as S, and undergoes a predefined maximum number of iterations, represented by MAX. The initial value of the logistic mapping is $Z_{0}$, which is equal to $Z_{0}\notin \{0.0,0.25,0.5,0.75,1.0\}$. The value designated as $Z_{i}$ is assigned to represent the value of the logistic mapping for the ith solution, and the range of values for i is {1,2,…,S}. The value of η is set to 4.

#### 3.1.2. Jellyfish Movement Patterns

1. Ocean current movement

The oceanic currents contain a substantial quantity of nutrients, which attracts jellyfish to follow these currents. The direction of the currents is determined by calculating the average vector position of each jellyfish in relation to the optimal jellyfish at its current location. This allows the jellyfish to identify the area with the optimal amount of food and to update its position accordingly.

(19) $$\vec{T}=Z_{best}-\beta \times rand(0,1)\times \mu$$

(20) $$Z_{i}(t+1)=Z_{i}(t)+rand(0,1)\times \vec{T}$$

In the context of oceanography, the variable $\vec{T}$ denotes the direction of the current, $Z_{best}$ represents the position of the optimal jellyfish within the current jellyfish swarm, β is the distribution coefficient, which is contingent upon the length of the current and assumes a value of 3, μ signifies the average position of all the jellyfish, and $Z_{i}$ and $Z_{i}(t+1)$ represent the locations of the jellyfish in the ith and i+1th generations, respectively, as they navigate through the flow of the current.

2. Jellyfish swarming movement

In the initial stages of formation, the majority of jellyfish within a swarm exhibit passive movement, maintaining their position while the swarm itself moves. This is evidenced by the observation that the jellyfish do not actively change their position but rather move around within their own area,

(21) $$Z_{i}(t+1)=Z_{i}(t)+rand(0,1)\times \gamma \times (U-L).$$

In this context, the coefficient of motion (denoted by γ) is a quantity greater than zero that is related to the length of the path traced by the jellyfish. It has a value of 0.1. The limits of the search space, designated as U and L, respectively, are also relevant for the purposes of this study.

As time progresses, the jellyfish demonstrates a tendency to move in the direction of its companions, who have located more food. This movement is manifested as an active movement, with the position updating the formula:

(22) $$Z_{i}(t+1)=Z_{i}(t)+rand(0,1)\times \vec{D}.$$

As time progresses, the jellyfish demonstrates an increasing capacity for independent action, influencing the overall direction of motion within the $\vec{D}$ jellyfish swarm.

(23) $$\vec{D}=\left\{\begin{array}{l} Z_{j}(t)-Z_{i}(t),if\;f(Z_{i})\geq f(Z_{j})\\ Z_{i}(t)-Z_{j}(t),if\;f(Z_{i})<f(Z_{j}) \end{array}\right.$$

In this context, $Z_{i}(t)$ signifies the location of the initial jellyfish during the tth iteration, whereas $Z_{i}(t)$ indicates a jellyfish chosen at random from the entire population.

#### 3.1.3. Time Control Mechanisms

The ocean currents that attract jellyfish are driven by the rich marine resources and comfortable living conditions that these creatures find in their environment. As time progresses, an increasing number of jellyfish congregate to form a swarm. When ocean currents change due to temperature or wind direction, the jellyfish in the swarm will move to another ocean current and form another swarm. The jellyfish within a swarm exhibit two distinct behavioural patterns: passive and active movements. Passive behaviour is the dominant form at the beginning, but as time passes, active behaviour becomes more and more dominant. This temporal control is achieved through the introduction of regulatory mechanisms that govern the movement of jellyfish in currents and within swarms.

A time control function and constant coefficients constitute the time control mechanism. The time control function is responsible for regulating the time control mechanism:

(24) $$C(t)=\left|\left(1-\frac{t}{MAX})\times (2\times rand(0,1)-1\right)\right|.$$

The value of C(t), which is dependent on the number of iterations, is subject to random variation within the range of 0 to 1. The starting point for the value of $C_{0}$ is set at 0.5, where t represents the current iteration count, and MAX denotes the maximum number of iterations that will be performed. In scenarios where C(t) is greater than or equal to $C_{0}$, the jellyfish adopt an active movement strategy by aligning themselves with the ocean currents. However, when C(t) is less than $C_{0}$, the jellyfish exhibit passive movement, confined to navigating solely within the confines of the jellyfish swarm.

#### 3.1.4. Algorithmic Process

The jellyfish search algorithm comprises two principal phases: search and exploitation. Initially, the probability of exploration exceeds that of exploitation, enabling the identification of a promising location. Over time, however, the probability of exploitation significantly surpasses that of exploration, allowing the jellyfish to locate the optimal position within the specified area.

(1) Initialisation: The initial position and velocity of the jellyfish must be set, as well as the adaptation value.
(2) Movement: The jellyfish’s position is recalculated or adjusted based on its current location and the speed and direction at which it is currently moving.
(3) Evaluation: The fitness value of each jellyfish should be calculated.
(4) Update optimal solution: The optimal solution is updated, both globally and for each individual.
(5) Synergistic behaviour: The speed of the jellyfish should be adjusted according to the current position and velocity through the utilisation of synergistic behaviour.
(6) Condition of termination: The algorithm will conclude its execution either when the predetermined number of iterations has been fully completed or if a specific stop condition has been met. If neither of these conditions is satisfied, the algorithm will proceed to repeat step (2) of the process.

### 3.2. Particle Swarm Algorithm

The particle swarm optimisation (PSO) algorithm is a computational method for identifying optimal solutions to a problem, which draws inspiration from the collective intelligence and foraging patterns observed in bird flocks. It was first proposed by Kennedy J and Eberthart R in 1995 [27].

The particle swarm optimisation (PSO) algorithm employs a set of massless particles to simulate the behaviour of a flock of birds foraging for food. The core properties of these particles are the velocity and position. The term “velocity” is used to describe the rate of movement, whereas “position” indicates the tendency of movement. In the context of a search, each particle independently searches for the optimal solution, designates it as the personal best, that is, the current individual extreme value, and disseminates it within the flock. By means of comparison, the optimal solution on a global scale, that is to say, the best of all the individual solutions, is established. Subsequently, all particles adjust their speed and position in an intelligent manner, with the objective of optimising the search path. This is based on their own individual extremes and the shared global optimal solution. This process is repeated until either a predefined number of iterations is reached, or the optimal solution is deemed to be sufficiently close.

In a D-dimensional search environment, N particles coalesce to form a cluster. Each particle, identified by the index i, is represented by a D-dimensional vector indicating its spatial position within the search space:

(25) $$X_{i}=(x_{i1},x_{i2},\ldots ,x_{iD})\;,\;i=1,2,\ldots ,N.$$

In order to represent the velocity, of the ith particle, the following equation can be used:

(26) $$V_{i}=(v_{i1},v_{i2},\ldots ,v_{iD})\;,\;i=1,2,\ldots ,N.$$

Furthermore, it is crucial to preserve the optimal solution, designated as $p_{best}$, that has been identified for each individual, as well as the optimal solution, designated as $g_{best}$, that has been identified for the entire cluster.

The velocity of each particle is adjusted or refreshed in accordance with the subsequent equation:

(27) $$v_{i}(t+1)=\omega \times v_{i}(t)+c_{1}\times r_{1}\times (p_{best\_i}-x_{i}(t))+c_{2}\times r_{2}\times (g_{best\_i}-x_{i}(t)).$$

In this context, the term ω represents the inertia weight, $v_{i}(t)$ denotes the velocity of particle i at the current moment t, $c_{1}$ and $c_{2}$ are the learning factors, $r_{1}$ and $r_{2}$ are random numbers within the range of [0, 1], $p_{best\_i}$ and $g_{best\_i}$ are the known individual optimal solution and the known population optimal solution, respectively, and $x_{i}(t)$ denotes the current position.

(28) $$x_{i}(t+1)=x_{i}(t)+v_{i}(t+1)$$

In the velocity update formulation, the inertia weights (ω) are initially employed to regulate the degree of influence exerted by the particle’s historical velocity on its current velocity. Then, the accelerations along the two directions, one directed by the individual’s best position ($p_{best\_i}$) and the other by the group’s best position ($g_{best\_i}$), are calculated independently. The degree of influence of these accelerations on the velocity is then controlled by the learning factors ($c_{1}$ and $c_{2}$). Finally, the values of $r_{1}$ and $r_{2}$ are used to increase the diversity and avoid premature convergence. The particle swarm optimisation method employs a simulation of the exploratory behaviour of particles within a solution domain, whereby the velocities and locations of the particles are iteratively refined. Each particle is guided by both the personal best position and the overall best position and thus progresses towards a more favourable solution. Upon completion of the iteration, the comprehensive cost function is optimised.

The fundamental operations of the algorithm are structured according to a defined sequence of steps, which are outlined as follows:

(1) The initialisation of a group of particles, including random positions and velocities, is undertaken.
(2) The fitness of each particle is then evaluated.
(3) A comparison is made between the current adaptation value and the previously recorded best position ($p_{best\_i}$) for each particle. If the current value is superior to the previous best, it becomes the new current best position ($p_{best\_i}$).
(4) For each particle, its adaptation value is evaluated in comparison to the global best position ($g_{best\_i}$) it has previously encountered. In the event that this value exceeds the current global best, the particle’s own best position is updated to align with the global best ($g_{best\_i}$).
(5) The particle’s velocity and location are updated in accordance with the prescribed velocity and position update equations.
(6) The algorithm terminates upon reaching the predefined number of iterations or satisfaction of the end condition. Otherwise, the process returns to step (2).

### 3.3. Genetic Algorithm

The origins of the genetic algorithm can be traced back to 1975, when it was first conceived by American academic J. Holland. In particular, the genetic algorithm is designed and proposed in accordance with the law of evolution observed in natural organisms, in other words, the principle of “survival of the fittest” applies. The fundamental principles of genetic algorithms are derived from Darwin’s theory of natural selection and are integrated with the essential concepts of genetics. In genetic algorithms, each solution instance is regarded as an “individual,” and the entire solution space constitutes a “population.” Each individual is represented by a string of “genes” that encode specific parameters of the solution. The quality of the population is enhanced through an iterative process, which approximates the optimal solution. The fundamental stages of the process include selection, crossover, and mutation [28].

Within the framework of genetic algorithms, the selection procedure is generally carried out utilising the subsequent formula:

(29) $$p_{i}=\frac{f(x_{i})}{\sum_{j=1}^{N}f(x_{i})}=\frac{f(x_{i})}{f_{sum}(x)}.$$

In this context, the term $f(x_{i})$ refers to the fitness of the ith individual, $f_{sum}(x_{i})$ represents the total fitness of the entire population, and $p_{i}$ denotes the probability of individual selection. One can posit that the likelihood of an individual being chosen for reproduction within a genetic algorithm is directly correlated with its fitness level.

(30) $$x_{new}=(x_{i1},x_{i2},\ldots ,x_{ik},x_{j(k+1)},\ldots ,x_{jn})$$

(31) $${x_{nk}}^{\prime }=x_{nk}+\delta$$

Equations (30) and (31) represent the crossover and mutation operations, respectively. The selected individuals generate new offspring through the process of crossover. If the crossover point is k, and two individuals, designated $x_{i}$ and $x_{j}$, are considered, then the resulting offspring is $x_{new}$. The mutation process entails the alteration of specific genes within newborn individuals at a minimal probability (μ), thereby fostering genetic variation and bolstering population diversity. In this context, the term δ represents the quantity of minor random variation.

The implementation of the algorithm is comprised of the following procedural steps:

(1) The initialisation of the population is the first step in the algorithm implementation. This involves the generation of an initial population, with each individual represented by a string of codes.
(2) Assess fitness: Each individual’s fitness is quantified, with those exhibiting superior fitness being more probable candidates for contributing to the subsequent generation.
(3) The selection process: A number of individuals exhibiting the greatest fitness are selected in preparation for crossover and mutation.
(4) Crossover operation: the selected individuals are paired in a manner that allows for the exchange of genetic material (e.g., single-point crossover).
(5) Mutation phase: With a minimal likelihood, selective alterations are introduced into individuals’ genes, fostering genetic variation and augmenting the population’s diversity.
(6) Generation of a new population: The formation of a new population is achieved by combining the most suitable individuals from the previous generation with those that have been generated anew.
(7) Termination condition: The iterative process reaches its conclusion when a predetermined ending condition is met, such as the attainment of a maximum iteration count or the achievement of a designated fitness level threshold.

### 3.4. Composite Improvement Algorithm

The composite improvement algorithm is a hybrid of the particle swarm, genetic, and jellyfish search algorithms. These three algorithms work in concert to identify the optimal solution. During the operation of the algorithm, the particle swarm algorithm continuously adjusts the speed and position of the particles in order to find a superior solution, based on both the individual’s historical optimal position and the global optimal position. Genetic algorithms, on the other hand, utilise selection, crossover, and mutation operations in order to identify superior adapted individuals within the population. The jellyfish search algorithm further explores the space through its following and searching behaviours.

The algorithm is comprised of the following principal steps:

(1) Initialisation phase

The initialisation phase comprises the definition of the search space, which encompasses the operational area of the unmanned aerial vehicle (UAV), as well as the identification of potential obstacles and target points.

The initialisation parameters encompass the parameters of the jellyfish search algorithm (swimming behaviour, foraging intensity, etc.), the parameters of the genetic algorithm (population size, crossover probability, mutation probability, etc.), and the parameters of the particle swarm algorithm (inertia weights, learning factors, etc.).

The initialisation phase involves the generation of an initial population with multiple path representations for the genetic and particle swarm algorithms.

The fitness function must then be set.

(2) Operation of the particle swarm algorithm: (1) Each individual should be randomly initialised with regard to both the velocity and position. (2) A comparison is made between the current particle fitness value and the historical optimal fitness value for that particle, with the individual optimal position ($p_{best}$) being updated in accordance with the result of this comparison. (3) The fitness value of each particle is then compared with the current global optimal value, and the global optimal position is updated accordingly. (4) The particle velocity is then updated in accordance with the individual’s historical optimal position, the global optimal position and the learning factor, which serves to update the particle velocity. (5) The particle position is then updated in accordance with the revised velocity.

(3) Operation of the genetic algorithm: (1) Following the application of the particle swarm algorithm, the population fitness value is utilised to select the optimal individual as a parent, employing techniques such as roulette selection. (2) The selected parent then undergoes crossover operations, including single-point crossover and multi-point crossover, to generate new individuals. (3) The newly generated individuals then undergo mutation operations.

(4) Perform jellyfish search algorithm operation: (1) A certain number of individuals follow the current global optimal individual with a probability. (2) The remaining individuals perform a random search to explore the new path space.

(5) Combined update: (1) The new individuals obtained after the three algorithm operations are combined. (2) The fitness value of each individual is recalculated. (3) The global optimal position and fitness value are then updated.

(6) Determine whether the maximum number of iterations has been reached. If this condition is met, conclude the iterative process. Otherwise, reiterate the execution of steps (2) to (5).

(7) Result output

The optimal path identified during the iterative process should be recorded, along with the corresponding fitness value.

**Figure 5:**
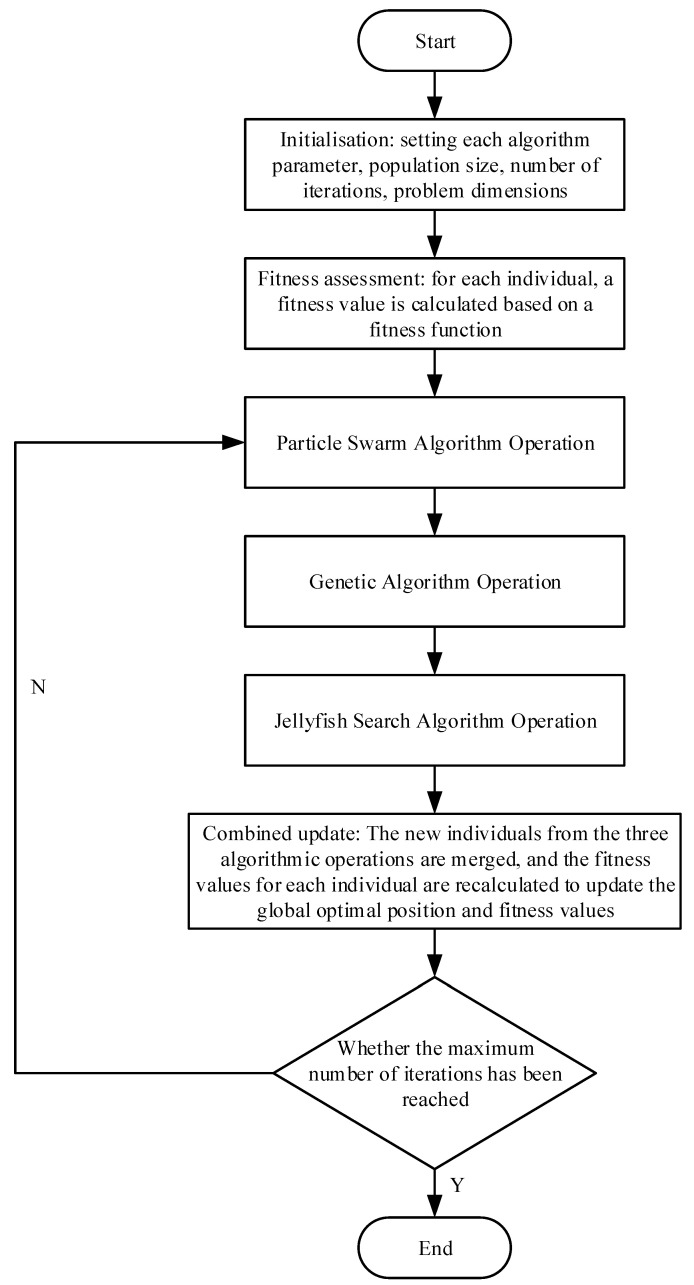
Flowchart of composite improvement algorithm.

The algorithmic flowchart and corresponding pseudo-code are presented in Figure 5 and Algorithm 1, respectively.
**Algorithm 1.** Pseudo-code for path planning algorithms.**Input**: Initial and target positions, obstacles, parameters (population size N, iterations T, crossover $p_{c}$, mutation $p_{m}$, learning factors $c_{1}$, $c_{2}$, etc.) **Output**: Optimal path 1: Initialise population $P=\left\{p_{1},p_{2},\ldots ,\;p_{N}\right\}$, set parameters2: **for** each individual $p_{i}$ in P **do**3:  Evaluate fitness $f(p_{i})$4: **end for**5: **for** iteration t = 1 to T **do**6:  **procedure** PSO7:   Update particle positions:8:   $v_{i}(t+1)=\omega \times v_{i}(t)+c_{1}\times r_{1}\times (p_{best\_i}-x_{i}(t))+c_{2}\times r_{2}\times (g_{best\_i}-x_{i}(t))$9:       $x_{i}(t+1)=x_{i}(t)+v_{i}(t+1)$10:  **end procedure**11:  **procedure** GA12:   Select parents based on fitness $f(p_{i})$, apply crossover:13:      $x_{new}=(x_{i1},x_{i2},\ldots ,x_{ik},x_{j(k+1)},\ldots ,x_{jn})$14:   Apply mutation:15:        ${x_{nk}}^{\prime }=x_{nk}+\delta$16:  **end procedure**17:  **procedure** JSO18:   Update position based on global best:19:    $Z_{i}(t+1)=Z_{i}(t)+rand(0,1)\times (Z_{best}-\beta \times rand(0,1)\times \mu )$20:   Or random swimming:21:     $Z_{i}(t+1)=Z_{i}(t)+rand(0,1)\times \gamma \times (U-L)$22:  **end procedure** 23:  Comprehensive update of the global best solution $p_{best}$ 24: **end for** 25: Return global best path

## 4. Simulation Experiment and Analysis

The experimental simulation framework outlined in this paper employed a 64-bit Windows 10 operating system powered by an Intel Core i7-14700F processor with a clock speed of 2.1 GHz, accompanied by 16 GB of RAM. The simulation tool adopted was MATLAB R2022b.

### 4.1. Test Function Simulation

In order to validate the efficacy and practicality of the algorithms introduced in this paper, a comparative analysis was conducted using five additional algorithms. The comparison included the following algorithms: the particle swarm optimisation algorithm, the genetic algorithm, the jellyfish search algorithm, the sparrow search algorithm, and the grey wolf optimisation algorithm. In order to substantiate the optimised and adaptive search capabilities of the enhanced composite algorithm, benchmark functions displaying diverse attributes from the CEC2005 dataset were employed for comparative evaluation against the aforementioned five algorithms. The performance of the algorithms was quantified in terms of the optimal value, mean value, and standard deviation across a series of benchmark functions. The optimal value serves to illustrate the algorithm’s capacity to identify solutions of the highest quality, thereby directly indicating its proficiency in attaining optimality. The mean value encapsulates the algorithm’s average performance over multiple trials and is, therefore, instrumental in assessing its stability and predictability. Lastly, the standard deviation quantifies the dispersion in results across iterations, thereby offering insights into the algorithm’s sensitivity to initial conditions and its overall robustness.

The CEC2005 function test set comprises 23 benchmark functions, which represent the most classical function test set currently in use. These functions have been verified in numerous academic publications [29]. The set includes seven single-peak benchmark functions ($F_{1}-F_{7}$), and the remaining 16 multi-peak benchmark functions ($F_{8}-F_{23}$) are included in the 23 benchmark functions. The dimensions of the first 13 functions are 30, 50, and 100, respectively, while the dimensions of the last 10 functions are fixed. A single-peak test function is defined as a function that contains a single global extreme point (typically the minimum point) and no other local extreme points within the domain of definition. This function is designed to specifically test the iterative convergence ability of the algorithm, that is to say, to assess whether the algorithm is capable of efficiently and accurately converging to the global optimal solution while requiring a relatively small number of iterations. Multi-peak test functions contain multiple local minima within the feasible domain, which often present a challenge to optimisation algorithms. The tendency of these algorithms is to converge toward a local optimum rather than achieving the global optimum. Therefore, the multi-peak test function requires that the algorithm be capable of discerning and escaping from the current local optimal area. Furthermore, the algorithm must have sufficient exploration ability to search in the global range and eventually find the true global optimal solution.

Table 1 presents the benchmark functions, where “dimension” denotes the dimensionality of the function set (specifically, the initial 13 benchmark functions are configured to operate in a 30-dimensional space), and “range” identifies the boundaries encompassing the search space of each function.

**Figure 6:**
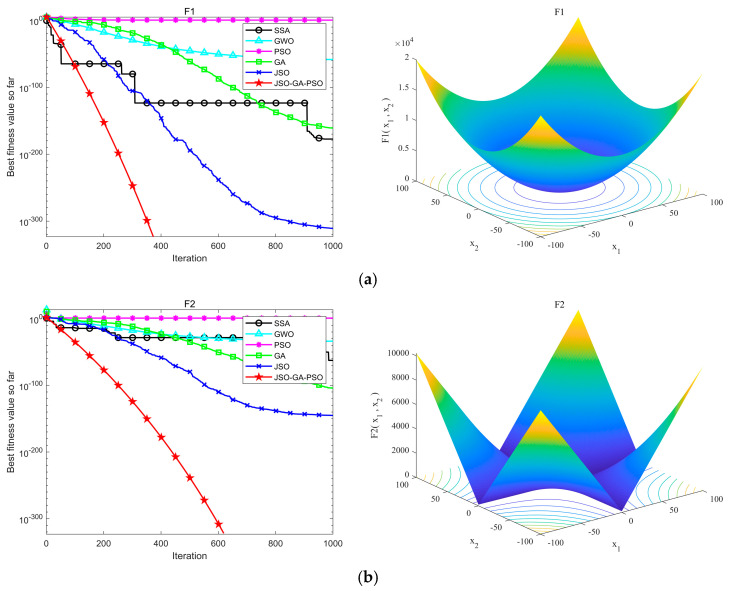

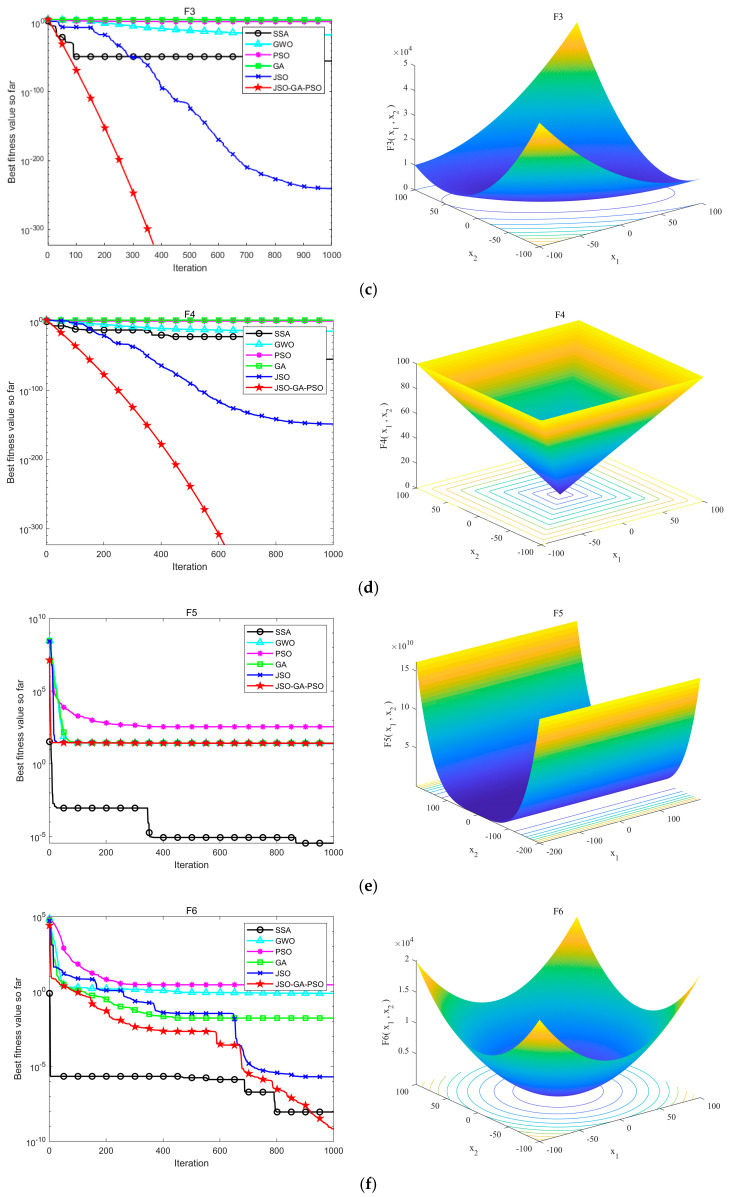

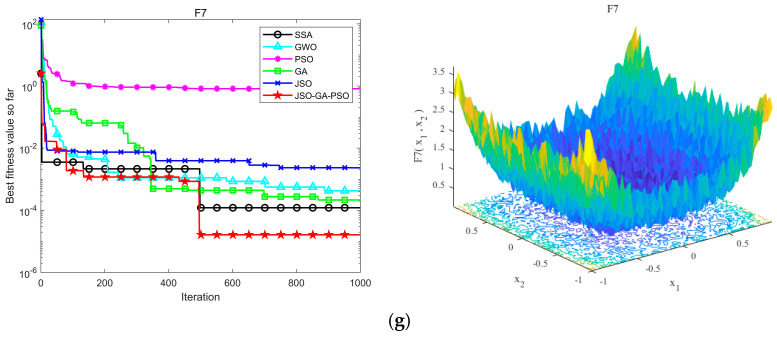
Single-peak function test results and function images, (**a**) F1, (**b**) F2, (**c**) F3, (**d**) F4, (**e**) F5, (**f**) F6, (**g**) F7.

In this experiment, a consistent population size of 30 was assigned, and the maximum iteration count was set to 1000. Each algorithm was executed 30 times to ensure consistency. The outcomes of these experimental tests are presented in Figure 5 and Figure 6. Table 2 offers a comparative analysis of the performance exhibited by the six algorithms across the various benchmark functions.

Figure 6 depicts the single-peak convergence process of each algorithm strategy comparison. A synthesis of the data and images in the table reveals that, in the $F_{1}$~$F_{4}$ single-peak test functions, the JSO-GA-PSO algorithm demonstrates optimal convergence precision, attaining the theoretical optimal solution with remarkable accuracy. In the $F_{4}$ single-peak test function, although the SSA algorithm exhibits a higher convergence accuracy than that of the JSO-GA-PSO algorithm, the JSO-GA-PSO algorithm exhibits a markedly accelerated convergence rate in comparison to the other algorithms under consideration. The algorithms achieve the optimal accuracy in a relatively short time. In the $F_{6}$, $F_{7}$ single-peak test functions, the JSO-GA-PSO algorithm demonstrates a clear advantage, not only in the convergence speed but also in the convergence theory and accuracy, reaching the theoretical optimum.

**Figure 7:**
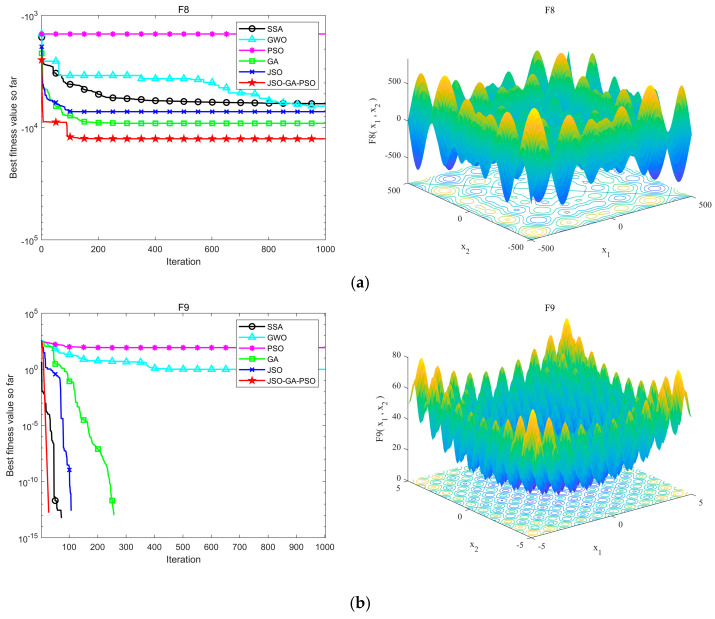

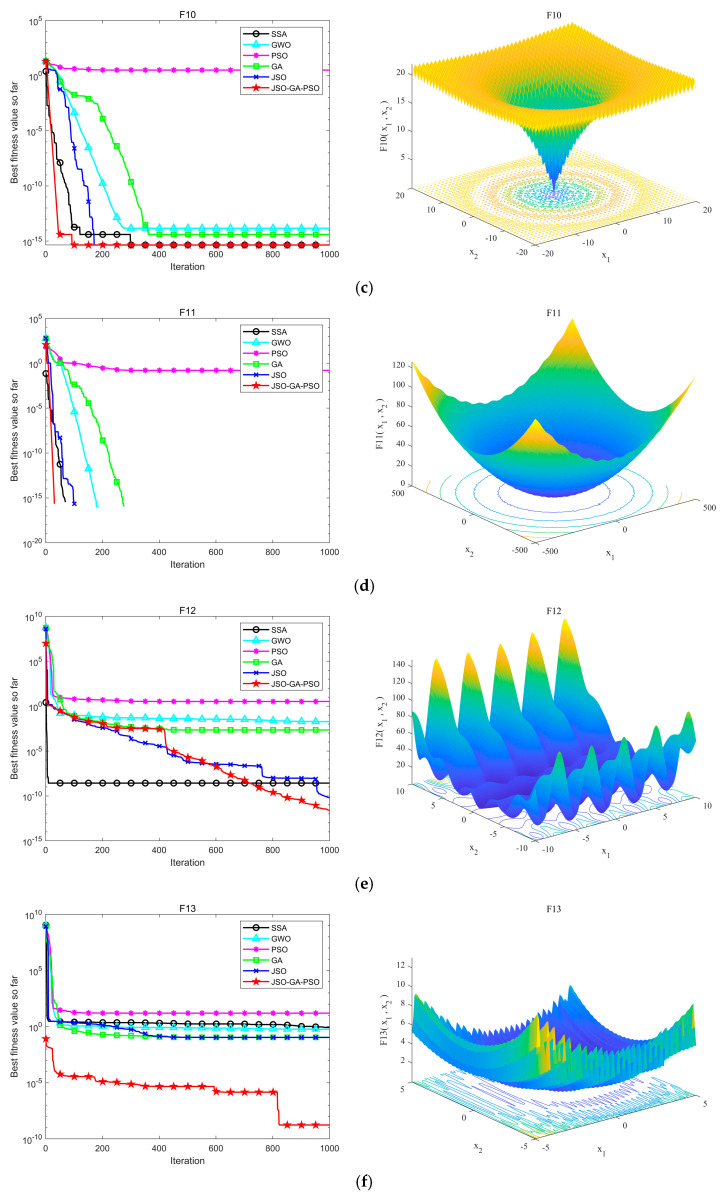

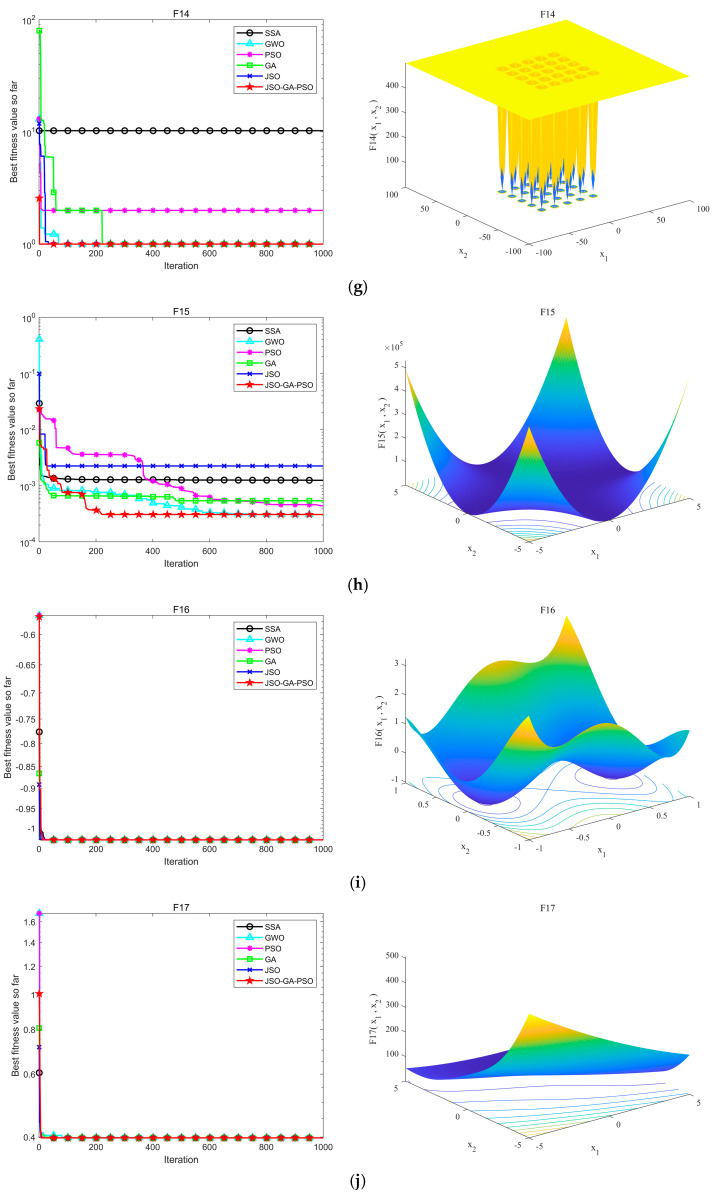

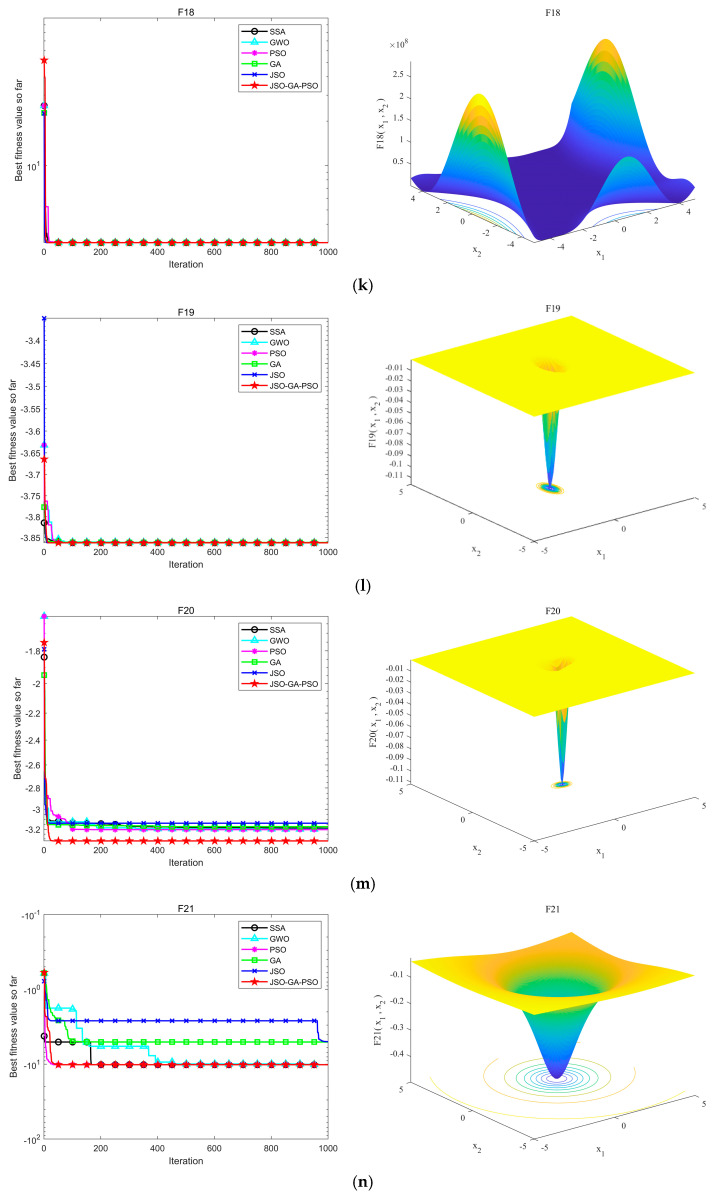

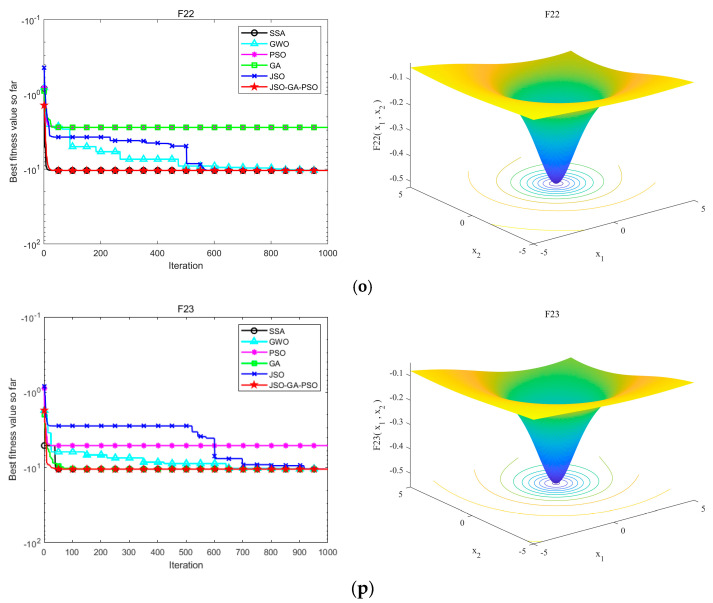
Multi-peak function test results and function images, (**a**) F8, (**b**) F9, (**c**) F10, (**d**) F11, (**e**) F12, (**f**) F13, (**g**) F14, (**h**) F15, (**i**) F16, (**j**) F17, (**k**) F18, (**l**) F19, (**m**) F20, (**n**) F21, (**o**) F22, (**p**) F23.

Figure 7 illustrates the multi-peak convergence trajectory of each algorithmic strategy. An examination of the accompanying data in the table allows us to deduce the following insights: In the $F_{8}$~$F_{11}$, $F_{13}$, $F_{15}$ multi-peak test functions, the JSO-GA-PSO algorithm demonstrates superior performance compared to other algorithms, achieving the highest level of convergence accuracy and the fastest convergence speed. In the $F_{6}$, $F_{12}$ multi-peak test functions, the JSO-GA-PSO algorithm exhibits the fastest convergence speed. In the $F_{14}$, $F_{16}$~$F_{23}$ multi-peak test functions, the JSO-GA-PSO algorithm also attains the highest convergence accuracy, reaching the desired accuracy in a relatively short period of time. Consequently, its convergence speed is significantly superior to that of the other algorithms.

**Table 2:** Test result statistics.

Function	Norm	SSA	GWO	PSO	GA	JSO	PSO-GA-JSO
$F_{1}(x)$	best	0	7.4146e-61	0.80032	2.2443e-168	2.8337e-313	**0**
	mean	3.2053e-41	5.2531e-59	2.9755	4.3489e-151	4.9223e-247	**0**
	std	1.7556e-40	1.3438e-58	1.3474	2.1571e-150	0	**0**
$F_{2}(x)$	best	0	6.5905e-36	3.2615	3.6411e-118	3.1648e-157	**0**
	mean	5.2727e-26	6.25e-35	6.5413	4.0055e-105	6.8735e-131	**0**
	std	2.0585e-25	5.9756e-35	1.7281	1.0782e-104	2.7133e-130	**0**
$F_{3}(x)$	best	0	2.293e-20	47.246	15,653.9066	4.1174e-264	**0**
	mean	1.963e-42	9.2572e-14	128.4404	20,202.5568	2.7769e-175	**0**
	std	1.0752e-41	3.4455e-13	58.1252	2827.8856	0	**0**
$F_{4}(x)$	best	0	7.041e-16	3.6029	0.017112	1.9357e-154	**0**
	mean	1.687e-26	1.467e-14	5.8227	36.9424	5.0824e-103	**0**
	std	8.5953e-26	1.0331e-14	1.2517	31.9573	2.7838e-102	**0**
$F_{5}(x)$	best	**4.5316e-08**	26.0638	185.2421	26.3872	24.602	24.6919
	mean	**1.1482e-05**	26.5972	731.5001	27.2173	24.9225	25.3091
	std	**1.5189e-05**	0.60775	567.5419	0.68742	0.17235	0.29882
$F_{6}(x)$	best	2.2568e-10	1.4778e-05	1.0787	0.012502	1.9613e-11	**3.2698e-13**
	mean	7.3256e-08	0.61549	3.1263	0.07243	3.7913e-08	**1.0418e-08**
	std	1.3147e-07	0.31105	1.1448	0.066829	1.1952e-07	**5.5817e-08**
$F_{7}(x)$	best	1.1246e-05	2.9383 e-04	0.11637	4.0421e-05	4.9554e-05	**1.2538e-07**
	mean	3.3045 e-04	8.3787 e-04	0.6341	0.0021085	7.6851 e-04	**2.2196 e-04**
	std	2.8183 e-04	5.5283 e-04	0.30112	0.0021384	4.7423 e-04	**2.1076 e-04**
$F_{8}(x)$	best	−12,569.4852	−7755.5984	−12569.4772	−12,569.4105	−12,537.16	−**3192.2498**
	mean	−8256.2513	−**6862.453**	−9095.0324	−10,458.7451	−8535.9335	−9095.0324
	std	1637.5937	462.3948	1370.7557	1995.334	2274.3265	**1.8501e-12**
$F_{9}(x)$	best	0	0	67.2969	0	0	**0**
	mean	0	0.49589	96.7162	0	0.47196	**0**
	std	0	1.4153	15.5094	0	2.3595	**0**
$F_{10}(x)$	best	4.4409e-16	1.1102e-14	3.2012	4.4409e-16	4.4409e-16	**4.4409e-16**
	mean	4.4409e-16	1.5484e-14	5.0506	4.4705e-15	6.8094e-16	**4.4409e-16**
	std	0	3.3227e-15	1.1044	2.234e-15	9.0135e-16	**0**
$F_{11}(x)$	best	0	0	0.086788	0	0	**0**
	mean	0	0.0022805	0.18908	0.0088319	0	**0**
	std	0	0.0059983	0.056399	0.02806	0	**0**
$F_{12}(x)$	best	1.0138e-12	3.7669e-06	1.3588	0.00052858	4.1989e-13	**3.7313e-14**
	mean	1.1977e-08	0.034059	4.1832	0.0090509	4.99e-05	**0.0034556**
	std	0.018927	0.016322	1.6624	0.01346	0.00027314	**2.2606e-08**
$F_{13}(x)$	best	1.9775e-10	0.10247	0.78417	0.026893	0.00026451	**1.0121e-13**
	mean	0.33142	0.51822	20.9477	0.18746	0.35597	**2.456e-07**
	std	0.30199	0.24689	17.08	0.14867	0.40219	**4.7772e-07**
$F_{14}(x)$	best	0.998	0.998	0.998	0.998	0.998	**0.998**
	mean	10.8244	3.0885	2.123	1.7535	**1.7523**	1.8218
	std	4.2116	3.5876	1.5783	1.8832	2.0366	**0.80773**
$F_{15}(x)$	best	0.00030749	0.00030749	0.00030749	0.00031084	0.00030749	**0.00030749**
	mean	0.00031198	0.00030782	0.0013529	0.00058106	0.00064559	**0.00030749**
	std	5.8467e-06	9.8269e-07	0.0036306	0.00017284	0.00029504	**2.1235e-19**
$F_{16}(x)$	best	−1.0316	−1.0316	−1.0316	−1.0316	−1.0316	−**1.0316**
	mean	−1.0316	−1.0316	−1.0316	−1.0316	−1.0316	−**1.0316**
	std	6.4883e-09	5.2775e-09	6.7752e-16	1.1866e-10	6.0459e-16	**6.4539e-16**
$F_{17}(x)$	best	0.39789	0.39789	0.39789	0.39789	0.39789	**0.39789**
	mean	0.39789	0.39789	0.39789	0.39789	0.39789	**0.39789**
	std	3.3636e-08	2.6292e-07	0	2.899e-06	0	**0**
$F_{18}(x)$	best	3	3	3	3	3	**3**
	mean	3	3	**3**	3	3	3.1
	std	1.2067e-07	6.4265e-06	**9.1458e-16**	5.2269e-05	1.9877e-15	4.9295
$F_{19}(x)$	best	−3.8628	−3.8628	−3.8628	−3.8628	−3.8628	−**3.8628**
	mean	−3.8628	−3.8621	−3.8628	−3.8611	−**3.8604**	−3.8617
	std	1.2456e-06	0.0019612	**2.6684e-15**	0.0018382	0.0036735	0.002725
$F_{20}(x)$	best	−3.2023	−3.322	−3.322	−**3.1963**	−3.322	−3.2031
	mean	−3.1914	−3.2134	−3.2715	−**3.145**	−3.2168	−3.2031
	std	0.0070232	0.053564	0.076441	0.035435	0.11767	**1.5026e-15**
$F_{21}(x)$	best	−10.1532	−10.1531	−10.1532	−10.1531	−10.1532	−**10.1531**
	mean	−**10.1532**	−9.4791	−5.2309	−8.3627	−6.4211	−5.4582
	std	**2.6318e-05**	1.7469	3.5679	2.8149	2.2875	2.5307
$F_{22}(x)$	best	−10.4029	−10.4029	−10.4029	−10.4024	−10.4029	−**10.4029**
	mean	−5.6577	−10.4024	−6.154	−7.4537	−7.4452	−**10.4029**
	std	2.5008	0.00031958	3.803	3.2981	3.0994	**3.1537e-05**
$F_{23}(x)$	best	−10.5364	−10.5364	−10.5364	−10.5363	−10.5364	−**10.5364**
	mean	−10.5364	−10.536	−6.5439	−10.3209	−9.1907	−**10.5364**
	std	1.8052e-05	0.00028867	3.0058	0.99717	2.5249	**2.4904e-15**

In conclusion, the JSO-GA-PSO algorithm demonstrates a notable superiority over the SSA, GWO, PSO, GA, and JSO algorithms, particularly in terms of the average optimisation search precision and the convergence velocity when evaluated on the test functions featuring a single peak. The results demonstrate that the JSO-GA-PSO algorithm is capable of effectively balancing global exploration with the ability to efficiently escape from local optimal solutions, thereby providing comprehensive validation of its robust capability in this regard. The performance of the JSO-GA-PSO algorithm on multi-peak test functions serves to illustrate its proficiency in exploring and locating the optimal solution, with results that surpass those of several renowned meta-heuristic algorithms across the majority of functions. Furthermore, the seamless transition between global and local exploration and exploitation strategies ensures a harmonious balance between the algorithm’s exploitation and exploration capabilities.

### 4.2. Path Planning Simulation

This section presents the validation of the proposed algorithm in the context of urban environments and potential threats. In order to simulate a complex urban environment, a map was created, which featured twenty buildings and two airborne threat zones. It should be noted that no flights were permitted at any altitude within the coordinates of the aforementioned building zones and threat zones. The dimensions of the simulation map were 10 km by 10 km by 0.25 km, the initial coordinates were (50, 900, 40), and the final coordinates were (950, 50, 0).

In order to assess the efficacy of the JSO-GA-PSO algorithm in the context of unmanned aerial vehicle (UAV) three-dimensional (3D) path planning, three additional algorithms were employed as benchmarks: the genetic algorithm (GA), particle swarm optimisation (PSO), and jellyfish swarm optimisation (JSO). The objective of this approach was to identify the limitations of the aforementioned algorithms, thus providing a robust basis for evaluating the JSO-GA-PSO algorithm’s capabilities. Each competitor commenced with a pool of 50 initial solutions and was subjected to a maximum of 100 iterations. Algorithm parameter settings are shown in Table 3.

The application of the GA algorithm yielded an optimal objective value of 38.8769, while the PSO algorithm resulted in 48.6766. The JSO algorithm returned 33.3629, and the JSO-GA-PSO algorithm produced 30.9275. In the absence of an optimisation algorithm, the optimal objective value was 178.2354.

**Figure 8:**
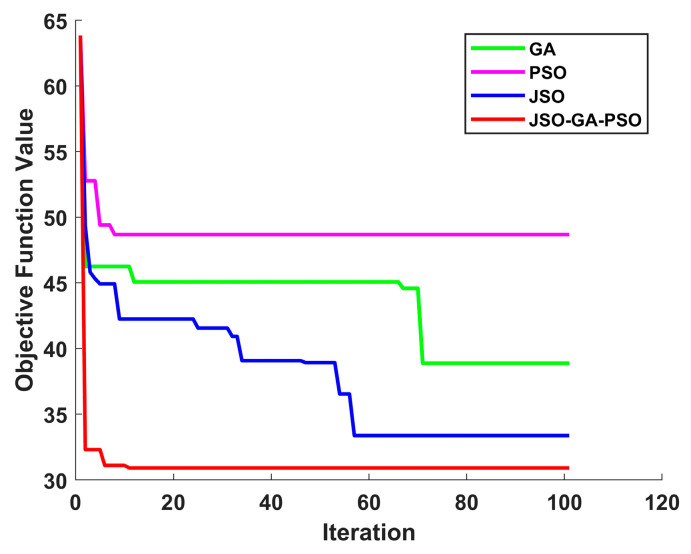
Objective function curve.

**Figure 9:**
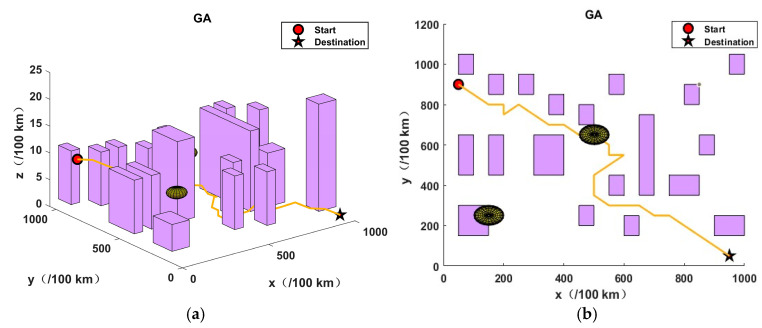
Representation of the drone in a 3D map using the GA algorithm, (**a**) front view, (**b**) top view.

**Figure 10:**
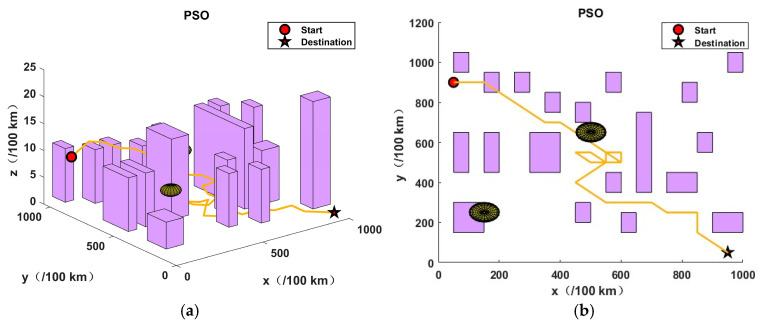
Representation of the drone in a 3D map using the PSO algorithm, (**a**) front view, (**b**) top view.

**Figure 11:**
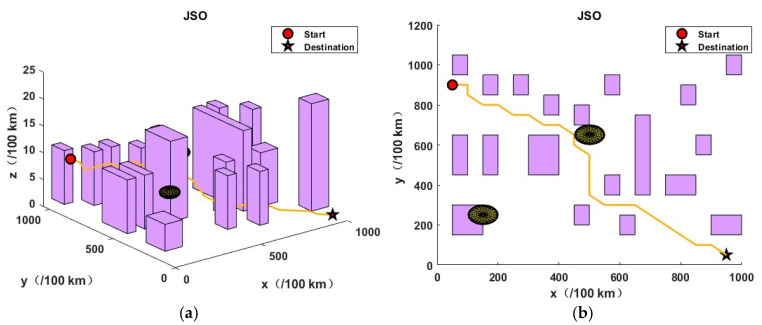
Representation of the drone in a 3D map using the JSO algorithm, (**a**) front view, (**b**) top view.

**Figure 12:**
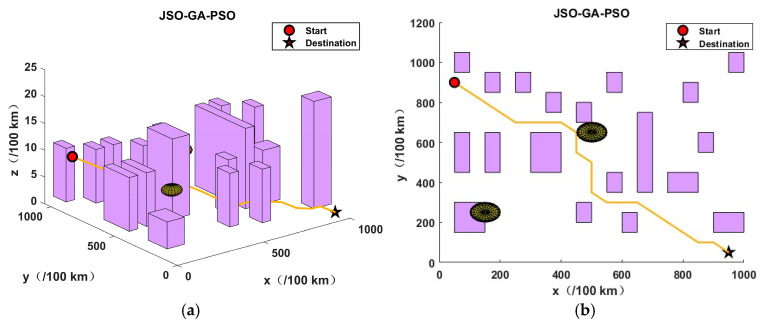
Representation of the drone in a 3D map using the JSO-GA-PSO algorithm, (**a**) front view, (**b**) top view.

**Figure 13:**
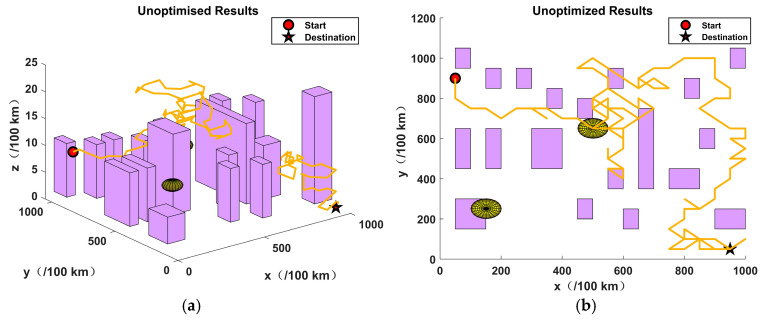
Representation of the drone in the 3D map without using the optimisation algorithm, (**a**) front view, (**b**) top view.

As illustrated in Figure 8, the results obtained by the enhanced composite algorithm after a limited number of iterations exceed those of the other three comparative algorithms, which required the full 100 iterations.The analysis presented in Figure 9, Figure 10, Figure 11 and Figure 12 illustrates the capacity of all four algorithms to devise a path traversing from the starting point to the destination within a complex three-dimensional environment. However, it can be observed that the GA, PSO, and JSO all exhibit varying degrees of local optimality. Conversely, the refined composite algorithm exhibits a significantly enhanced capability to circumvent local optima, resulting from the integration of augmented search behaviour and cross variation as a disruptive force. This outcome further corroborates the enhanced optimality-seeking ability of the JSO-GA-PSO algorithm, which is reflected in the improved composite algorithm’s ability to plan a path with a shorter length and a higher quality. Figure 13 shows the unoptimised results.

In conclusion, the enhanced composite algorithm, when contrasted with the GA, PSO, and JSO, evinces a discernible enhancement in overall performance. Primarily, it attains an equilibrium between global and local search capabilities, rendering it especially suited for addressing the UAV 3D path planning challenge. Secondly, it augments the algorithm’s capacity to transcend local optima, ensuring the final output is in close approximation to the global optimum while effectively circumventing premature convergence.

In order to eliminate potential errors and validate the reliability of the enhanced composite algorithm, this study conducted 100 simulations, comparing the four algorithms. The findings demonstrate that the JSO-GA-PSO algorithm consistently produces fitness values between 30.9 and 31, while the JSO algorithm stabilises at a value between 33 and 34. Similarly, the PSO algorithm demonstrates stability within the range of 48.5 to 49.8, while the GA algorithm exhibits fluctuations between 38.7 and 39.8. It is noteworthy that the intervals of the comparison algorithms’ fitness values are significantly broader than that of the improved composite algorithm. By presenting the fitness values to four decimal places, the results are more clearly discernible. Consequently, the refined composite algorithm exhibits greater resilience against algorithmic performance variability due to chance, demonstrating the most consistent path planning capability compared to the other three algorithms.

**Figure 14:**
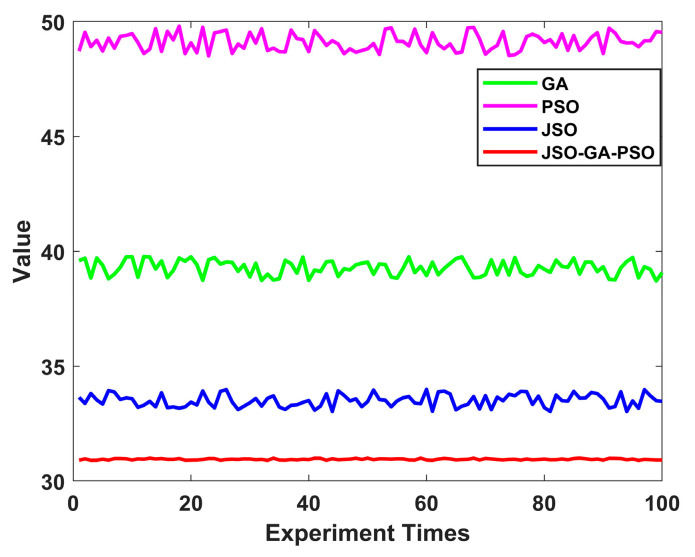
The results of repeating the experiment 100 times.

A smaller mean value indicates a lower aggregate value of the data, while a lower variance signifies a tighter distribution of the data. Figure 14 shows a statistical graph of the results of 100 experiments. As evidenced in Table 4, the mean adaptation values for the GA, PSO, JSO, and JSO-GA-PSO algorithms are 39.2261, 49.1558, 33.4675, and 30, respectively. The value of 9528 indicates that the improved composite algorithm is capable of planning the shortest and most effective obstacle avoidance path. Secondly, the adaptive variance of the GA algorithm is 0.32, the adaptive variance of the PSO algorithm is 0.3574, the adaptive variance of the JSO algorithm is 0.2897, and the adaptive variance of the JSO-GA-PSO algorithm is 0.0297. This indicates that the improved composite algorithm has enhanced stability in optimisation-seeking.

Furthermore, this paper employs Monte Carlo methods to generate input conditions, verifying the algorithm’s robustness. In particular, the coordinates of the initial point are generated through the utilisation of random numbers within a specified range. To prevent the generation of starting coordinates that are in close proximity to the end coordinates, a restriction is placed on the range of coordinates that can be generated. This is completed to ensure the effectiveness of the algorithm, as proximity between the two sets of coordinates could otherwise render the algorithm ineffective. Furthermore, the sizes of the two airborne obstacles are modified using random numbers to reflect the potential inaccuracies in the identification of barriers by the UAV sensors. The experiment was conducted ten times, and the results are shown in Table 5.

In a complex and uncertain environment, the improved composite algorithm generates stable results and still exhibits the optimal path planning outcomes, fully demonstrating the robustness of the algorithm. The randomly generated starting point coordinates present a more rigorous challenge for path planning. Meanwhile, for the two airborne obstacles, their sizes are no longer fixed values; instead, random numbers are employed to generate corresponding perturbations. The introduction of these perturbations ingeniously simulates the error scenarios existing in the process of obstacle identification by UAV sensors, highly reproducing the complex conditions that may arise during actual flight situations.

## 5. Conclusions

This paper presented a novel and refined composite algorithm dedicated to the optimisation of three-dimensional trajectories for unmanned aerial vehicles (UAVs). The proposed algorithm incorporates several enhancements to improve its performance. Firstly, a logical chaos mapping technique was introduced to enhance the algorithm’s capacity to explore a diverse range of solutions. Secondly, the jellyfish algorithm was integrated into the particle swarm algorithm, thereby further enhancing the versatility and resilience of the algorithm. Thirdly, the crossover and mutation mechanisms derived from the genetic algorithm were incorporated to enhance the diversity of the algorithmic population, while the element of randomness was introduced. The experimental results demonstrate that the enhanced composite algorithm proposed in this paper markedly enhances the efficiency of unmanned aerial vehicle (UAV) 3D spatial path planning. This is evidenced by the algorithm’s capacity to integrate global search capabilities at the initial stage of the iteration and to combine local search capabilities at subsequent stages. This enables the algorithm to design high-quality flight paths. Furthermore, the enhanced composite algorithm exhibits enhanced and more robust optimisation capabilities, which effectively prevent premature maturation or local optimal traps. This ensures that the final output is as close as possible to the global optimal solution, overcoming the limitations of a single algorithm and allowing the hybrid algorithm to fully utilise its advantages. The quality and efficiency of path planning are improved, and the algorithm provides strong support for the safe and efficient execution of UAV missions. The aforementioned conclusions serve to illustrate that the enhanced composite algorithm is capable of path planning in complex urban environments, a capability of considerable practical significance.

The algorithm exhibits certain boundaries in engineering applications, yet also holds the feasibility of practical application within existing sensor networks. Concerning engineering boundaries, the limitations of sensor accuracy and the dynamic environmental changes lead to restrictions on the algorithm in environmental perception and modelling. Additionally, the complexity of the algorithm may collide with the limited computational resources of unmanned aerial vehicles (UAVs), thereby influencing real-time performance. Nevertheless, from the perspective of feasibility, the diverse and distributed arrangement of sensors in the current sensor network can supply abundant and comprehensive data for the algorithm to bolster path planning. Furthermore, with the application of edge computing and the progress of communication technology, both data-processing and communication capabilities are strengthened, which can reduce latency. Moreover, through improvement measures like lightweight optimisation or combination with other algorithms, the performance of the algorithm within sensor networks can be further enhanced to better accommodate the actual engineering requirements.

The next phase will be based on this work, to conduct further research on path planning in unknown environments and multi-machine cooperative path planning. It is anticipated that a convergence of classical and intelligent algorithms will be employed to reinforce the system’s stability and overall cluster feasibility in multi-machine collaboration scenarios, thereby enhancing the system’s robustness.

## Figures and Tables

**Table 1:** Benchmark functions.

Function	Dim	Limit
$F_{1}(x)=\sum_{i=1}^{n}x_{i}^{2}$	30	[−100, 100]
$F_{2}(x)=\sum_{i=1}^{n}\left|x_{i}\right|+\prod_{i=1}^{n}\left|x_{i}\right|$	30	[−10, 10]
$F_{3}(x)={\sum_{i=1}^{n}\left(\sum_{j=1}^{i}x_{i}\right)}^{2}$	30	[−100, 100]
$F_{4}(x)=\max_{i}\{\left|x_{i}\right|,1\leq i\leq n\}$	30	[−100, 100]
$F_{5}(x)=\sum_{i=1}^{n}\left[100{\left(x_{i+1}-x_{i}^{2}\right)}^{2}+{\left(x_{i}-1\right)}^{2}\right]$	30	[−30, 30]
$F_{6}(x)=\sum_{i=1}^{n}{\left([x_{i}+0.5]\right)}^{2}$	30	[−100, 100]
$F_{7}(x)=\sum_{i=1}^{n}x_{i}^{4}+random[0,1)$	30	[−128, 128]
$F_{8}(x)=\sum_{i=1}^{n}-x_{i}\sin(\sqrt{\left|x_{i}\right|})$	30	[−500, 500]
$F_{9}(x)=\sum_{i=1}^{n}\left[x_{i}^{2}-10\cos(2\pi x_{i})+10\right]$	30	[−5.12, 5.12]
$F_{10}(x)=-20\exp(-0.2\sqrt{\frac{1}{n}\sum_{i=1}^{n}x_{i}^{2}})-\exp(\frac{1}{n}\sqrt{\sum_{i=1}^{n}\cos(2\pi x_{i})})+20+e$	30	[−32, 32]
$F_{11}(x)=\frac{1}{4000}\sum_{i=1}^{n}x_{i}^{2}-\prod_{i=1}^{n}\cos(\frac{x_{i}}{\sqrt{i}})+1$	30	[−50, 50]
$\begin{array}{cc} F_{12}(x)= & \frac{\pi }{n}\{10\sin(\pi y_{1})+\sum_{i=1}^{n}{\left(y_{i}-1\right)}^{2}{\left[1+10\sin^{2}(\pi y_{i+1})\right]}^{2}\}+\\ & \sum_{i=1}^{n}u(x_{i},10,100,4)\;y_{i}=1+\frac{x_{i}+1}{4} \end{array}$	30	[−50, 50]
$\begin{array}{cc} F_{13}(x)= & 0.1\{\sin^{2}(3\pi x_{1})+\sum_{i=1}^{n}{\left(x_{i}-1\right)}^{2}[1+\sin^{2}(3\pi x_{i}+1)]+\\ & {\left(x_{n}-1\right)}^{2}[1+\sin^{2}(2\pi x_{n})]\}+\sum_{i=1}^{n}u(x_{i},5,100,4) \end{array}$	30	[−50, 50]
$F_{14}\left(x\right)={\left(\frac{1}{500}+\sum_{j=1}^{25}\frac{1}{j+\sum_{i=1}^{2}{\left(x_{i}-a_{ij}\right)}^{6}}\right)}^{-1}$	2	[−65, 65]
$F_{15}(x)=\sum_{i=1}^{11}{\left[a_{i}-\frac{x_{1}(b_{i}^{2}+b_{i}x_{2})}{b_{i}^{2}+b_{i}x_{3}+x_{4}}\right]}^{2}$	4	[−5, 5]
$F_{16}(x)=4x_{1}^{2}-2.1x_{1}^{4}+\frac{1}{3}x_{1}^{6}+x_{1}x_{2}-4x_{2}^{2}+4x_{2}^{4}$	2	[−5, 5]
$F_{17}(x)={\left(x_{2}-\frac{5.1}{4\pi^{2}}x_{1}^{2}+\frac{5}{\pi }x_{1}-6\right)}^{2}+10(1-\frac{1}{8\pi })\cos x_{1}+10$	2	[−5, 5]
$\begin{array}{cc} F_{18}(x)= & [1+{\left(x_{1}+x_{2}+1\right)}^{2}(19-14x_{1}+3x_{1}^{2}-14x_{2}+6x_{1}x_{2}+3x_{2}^{2})]\times \\ & \left[30+{\left(2x_{1}-3x_{2}\right)}^{2}\times (18-32x_{1}+12x_{1}^{2}+48x_{2}-36x_{1}x_{2}+27x_{2}^{2})\right] \end{array}$	2	[−2, 2]
$F_{19}(x)=\sum_{i=1}^{4}c_{i}\exp(-\sum_{j=1}^{3}a_{ij}{\left(x_{i}-p_{ij}\right)}^{2})$	3	[0, 1]
$F_{20}(x)=\sum_{i=1}^{4}c_{i}\exp(-\sum_{j=1}^{6}a_{ij}{\left(x_{i}-p_{ij}\right)}^{2})$	6	[0, 1]
$F_{21}(x)=-\sum_{i=1}^{5}{\left[(X-a_{i}){\left(X-a_{i}\right)}^{T}+c_{i}\right]}^{-1}$	4	[0, 10]
$F_{22}(x)=-\sum_{i=1}^{7}{\left[(X-a_{i}){\left(X-a_{i}\right)}^{T}+c_{i}\right]}^{-1}$	4	[0, 10]
$F_{23}(x)=-\sum_{i=1}^{10}{\left[(X-a_{i}){\left(X-a_{i}\right)}^{T}+c_{i}\right]}^{-1}$	4	[0, 10]

**Table 3:** Algorithm parameter setting.

$p_{1}$	$p_{2}$	ω	$c_{1}$	$c_{2}$
0.85	0.1	0.1	1	1

**Table 4:** Statistics of 100 simulation experiments.

	Mean Value of Adaptation	Adaptation Variance
GA	39.2808	0.3267
PSO	49.1077	0.3766
JSO	33.5044	0.2749
JSO-GA-PSO	30.9478	0.0291

**Table 5:** Statistics of simulation experimental results of Monte Carlo method with random inputs.

	GA	PSO	JSO	JSO-GA-PSO	Unoptimised Results
1	33.7771	35.2412	27.9275	**26.2665**	126.0127
2	43.2911	38.1701	36.5091	**31.4381**	199.5736
3	32.9487	33.8236	30.3953	**29.2419**	186.3994
4	37.0484	34.9487	30.1667	**29.1455**	365.2738
5	32.6308	34.0450	27.8465	**26.6349**	191.2565
6	36.0450	33.4592	30.3846	**29.5597**	460.0597
7	31.2166	31.1168	28.1920	**27.3636**	149.1616
8	40.3874	37.5091	32.7394	**30.0990**	180.5710
9	41.9233	37.7308	30.9487	**28.1701**	307.8618
10	35.3629	33.9022	28.5344	**27.0737**	137.4050

## Data Availability

Data are contained within the article.
